# Sex‐specific regulation of aging in *Caenorhabditis elegans*

**DOI:** 10.1111/acel.12724

**Published:** 2018-03-01

**Authors:** Bernadette Hotzi, Mónika Kosztelnik, Balázs Hargitai, Krisztina Takács‐Vellai, János Barna, Kincső Bördén, András Málnási‐Csizmadia, Mónika Lippai, Csaba Ortutay, Caroline Bacquet, Angela Pasparaki, Tamás Arányi, Nektarios Tavernarakis, Tibor Vellai

**Affiliations:** ^1^ Department of Genetics Eötvös Loránd University Budapest Hungary; ^2^ Department of Biological Anthropology Eötvös Loránd University Budapest Hungary; ^3^ Department of Biochemistry Eötvös Loránd University Budapest Hungary; ^4^ Department of Anatomy, Cell‐ and Developmental Biology Eötvös Loránd University Budapest Hungary; ^5^ HiDucator Ltd Kangasala Finland; ^6^ Institute of Enzymology Research Centre for Natural Sciences Hungarian Academy of Sciences Budapest Hungary; ^7^ Institute of Molecular Biology and Biotechnology Foundation for Research and Technology‐Hellas Heraklion Greece; ^8^ BNMI (INSERM 1083/CNRS 6214) Université d'Angers Angers France; ^9^ MTA‐ELTE Genetics Research Group Eötvös Loránd University Budapest Hungary

**Keywords:** aging, *Caenorhabditis elegans*, *daf‐16/FOXO*, dauer development, insulin/IGF‐1 signaling, sex determination, TRA‐1/GLI

## Abstract

A fascinating aspect of sexual dimorphism in various animal species is that the two sexes differ substantially in lifespan. In humans, for example, women's life expectancy exceeds that of men by 3–7 years. Whether this trait can be attributed to dissimilar lifestyles or genetic (regulatory) factors remains to be elucidated. Herein, we demonstrate that in the nematode *Caenorhabditis elegans*, the significantly longer lifespan of hermaphrodites—which are essentially females capable of sperm production—over males is established by TRA‐1, the terminal effector of the sex‐determination pathway. This transcription factor directly controls the expression of *daf‐16/FOXO*, which functions as a major target of insulin/IGF‐1 signaling (IIS) and key modulator of aging across diverse animal phyla. TRA‐1 extends hermaphrodite lifespan through promoting *daf‐16* activity. Furthermore, TRA‐1 also influences reproductive growth in a DAF‐16‐dependent manner. Thus, the sex‐determination machinery is an important regulator of IIS in this organism. These findings provide a mechanistic insight into how longevity and development are specified unequally in the two genders. As TRA‐1 is orthologous to mammalian GLI (glioma‐associated) proteins, a similar sex‐specific mechanism may also operate in humans to determine lifespan.

## 1 INTRODUCTION

A remarkable phenomenon in aging biology is that the two genders display significantly different lifespans in divergent, sexually dimorphic animal species. For example, in flies, mice, and humans, females have a tendency to live longer than males (in human populations, the lifespan advantage of women over men can achieve up to 7–8 years; Blagosklonny, 2010; Eskes & Haanen, 2007; Gems, 2014; La Croix et al.*,* 1997; Lints, Bourgois, Delalieux, Stoll & Lints, 1983; Tower, 2006; Tower & Arbeitman, 2009; Vina, Borrás, Gambini, Sastre & Pallardó, 2005). In these species, the heterogametic sex (XY) is male. In contrast, in species where the heterogametic sex (ZW) is female (e.g., in most bird species), males tend to live longer than females. Moreover, genetic and environmental factors that influence lifespan often have a larger effect in one sex than the other (Partridge, Gems & Withers, 2005). The question whether sex‐specific differences in lifespan are determined by genetic regulatory mechanisms or are merely the by‐products of different lifestyles (e.g., males are generally more predisposed than females to engage in fights) remains a great challenge for science, one with significant medical and social implications (Blagosklonny, 2010).

The nematode *Caenorhabditis elegans* develops as either a male having only one sex chromosome (XO) in the somatic cells or a hermaphrodite with two sex chromosomes (XX) in the somatic cells (the self‐fertile hermaphrodite is essentially a female that produces sperm for a brief period before oogenesis; Zarkower, 2006). Gametes with no sex chromosome are generated as a consequence of chromosome nondisjunction during meiosis which is a rather rare event; in wild‐type populations, males present only at low (~0.3%) frequency. Somatic sexual fates in *C. elegans* are specified by the global sex‐determination pathway, the terminal effector of which is the transcription factor TRA‐1 (sexual transformer) that exists in two major isoforms, A and B (Zarkower & Hodgkin, 1992). TRA‐1 is orthologous to mammalian GLI (glioma‐associated) and *Drosophila* Ci (Cubitus interruptus) proteins and determines hermaphrodite features by repressing the expression of male‐specific genes (Berkseth, Ikegami, Arur, Lieb & Zarkower, 2013; Chen & Ellis, 2000; Conradt & Horvitz, 1999; Hargitai et al., 2009; Mason, Rabinowitz & Portman, 2008; Schwartz & Horvitz, 2007; Szabó et al., 2009; Yi, Ross & Zarkower, 2000). In males, TRA‐1 appears to play only a minor role (Schvarzstein & Spence, 2006). *tra‐1* transcription is most active during embryonic development (Zarkower & Hodgkin, 1992). At adult stages, a TRA‐1A variant, TRA‐1^100^, which is a C‐terminally truncated form of TRA‐1A isoform, accumulates at much higher levels in hermaphrodites than in males, and this sex‐specific difference appears to result from the proteolytic degradation of TRA‐1^100^ in males (Schvarzstein & Spence, 2006; Starostina et al., 2007).

In mixed *C. elegans* populations containing both sexes, hermaphrodites significantly outlive males (Gems & Riddle, 1996; Johnson & Hutchinson, 1993; Johnson & Wood, 1982). Lifespan advantage in hermaphrodites disappears when animals are grown individually or unable to physically interact with each other: the lifespan of solitary or paralyzed males is nearly 30% longer than that of isolated or grouped hermaphrodites (Gems & Riddle, 2000). In the absence of hermaphrodites, however, males frequently leave the area of food source (*Escherichia coli* bacteria) to find a mating partner―this phenomenon is called mate‐searching behavior (Lipton, Kleemann, Ghosh, Lints & Emmons, 2004)―and males leaving the bacterial layer are subjected to calorie restriction or intermittent/prolonged starvation. Both conditions are known to extend lifespan significantly in various animal species (Koubova & Guarente, 2003). It is worth noting that severely paralyzed nematodes that were previously placed onto the bacterial layer consume bacterial cells nearby their body, thereby also becoming starved by time. Another study on mixed *C. elegans* populations showed recently that males shorten the lifespan of hermaphrodites via secreted pheromones (Maures et al., 2014). In the experimental design, the analysis applied a nearly equal number of males and hermaphrodites were assayed on each test plate (200‐200 hermaphrodites and males/plate). Under these circumstances, hermaphrodites did not display a longevity advantage over males, rather the two genders lived almost identical long. The 1:1 sex ratio and relatively high population density however are quite far from that observed in nature. So, free‐living hermaphrodite animals are likely not to be exposed to such high doses of male substances, and their lifespan is probably less significantly affected by the opposite sex. In addition, both mating and male pheromone, although through distinct mechanisms, shorten lifespan in males (Shi, Runnels & Murphy, 2017). The former factor also limits lifespan in hermaphrodites (Shi & Murphy, 2014). Thus, many aspects of sexual interaction strongly affect the lifespan of both sexes in this organism.

The use of mutations in key sex‐determination genes revealed that the presence of two X chromosomes restricts hermaphrodite lifespan (Hartman & Ishii, 2007). Expression data of autosomal and X chromosome‐linked genes suggested that the level of dosage compensation (this mechanism equalizes the expression of X chromosome‐linked genes between the two sexes) declines as the hermaphrodite animal ages. Age‐related decrease in dosage compensation may limit lifespan in XX animals. Together, the aging process is determined unequally in the two *C. elegans* sexes, and lifespan regulation occurs in a complex way that involves different environmental, behavioral, and genetic factors, including the dosage compensation machinery.

In this work, we aimed to culture nematodes under conditions that approximate to those found in their natural environments (relatively low population density and male scarcity). Under these settings, hermaphrodites lived significantly longer than males. We also found that increased longevity in hermaphrodite animals depends on the nematode sex‐determination pathway and that this regulatory gene cascade influences the activity of the Forkhead‐like transcription factor DAF‐16 (dauer formation defective), the effector of insulin/IGF‐1 (insulin‐like growth factor 1) signaling (IIS). Thus, IIS, which regulates aging across divergent animal phyla, is adjusted unevenly between the two genders in this organism and perhaps in other animal species.

## 2 RESULTS

### 2.1 Hermaphrodites live longer than males in mixed *C. elegans* populations with hermaphrodite abundance

**Figure 1:**
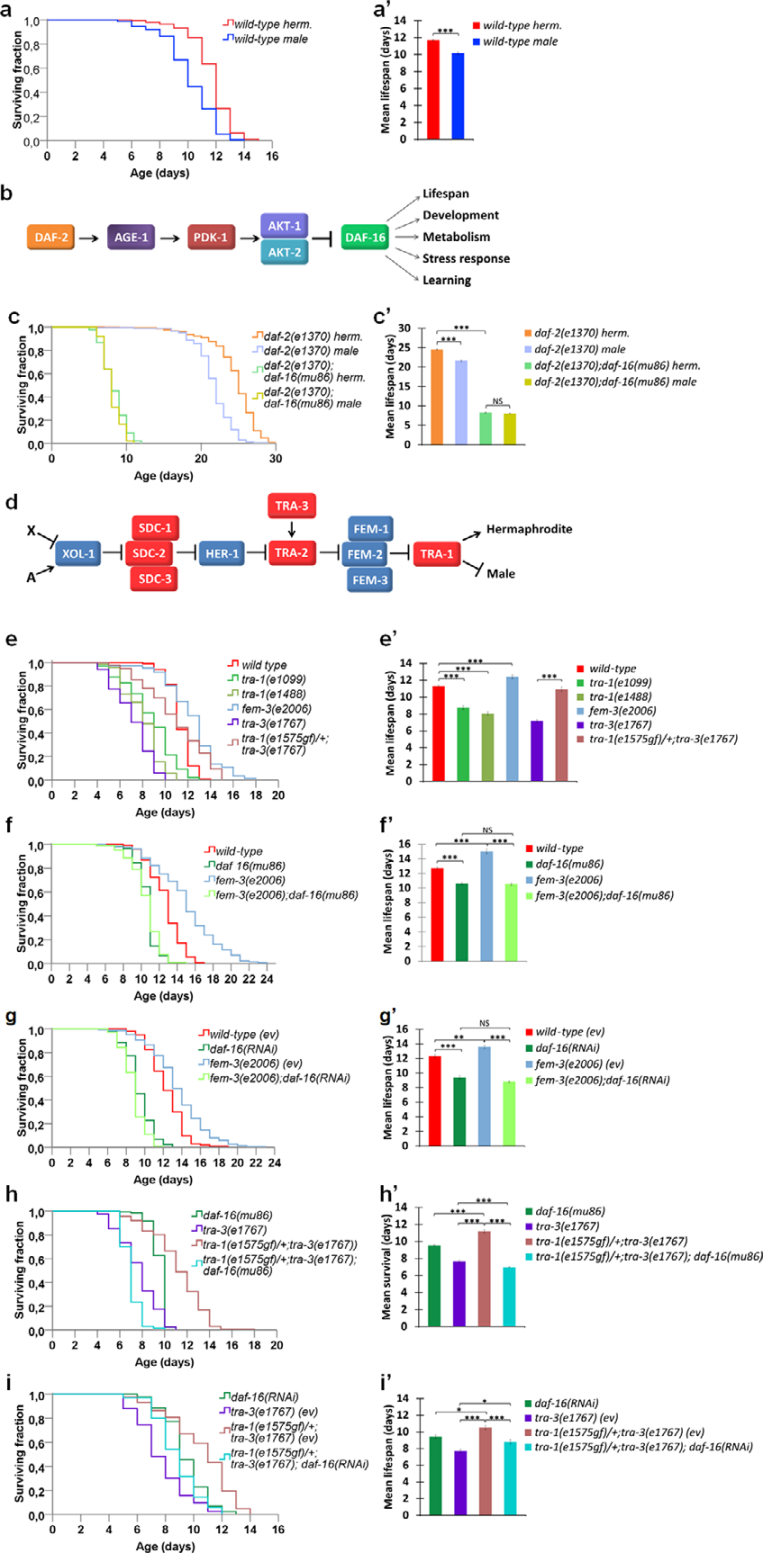
The terminal sex‐determining factor TRA‐1 promotes hermaphrodite longevity by enhancing *daf‐16* activity. (a, a') When maintained in groups containing both sexes, and with a great majority of hermaphrodite animals, wild‐type hermaphrodites (red curve) live significantly longer than males (blue curve). (b) The insulin/IGF‐1 signaling pathway in *C. elega*ns. DAF‐2: insulin/insulin‐like growth factor receptor 1; AGE‐1: type I phosphatidylinositol‐3‐kinase; PDK‐1: phosphoinositide‐dependent kinase; AKT‐1, ‐2: AKT/PKB‐AKT8 virus protooncogene/protein kinase B; DAF‐16: FOXO‐like transcription factor. (c, c') In *daf‐2(‐)* mutant background, the longer lifespan of hermaphrodites over males depends on *daf‐16* activity. Animals were maintained at 20°C until they developed into the L4 larval stage and then transferred at 25°C. (d) The *C. elegans* sex‐determination cascade. TRA‐1, the terminal effector of the cascade, is essentially active only in hermaphrodites but not in males. X: sex chromosome; A: autosome. (e, e') Inactivation of *tra‐1* decreases while inactivation of *fem‐3* (which corresponds to hyperactive *tra‐1*) increases lifespan. A *tra‐1* gain‐of‐function (gf) mutation, *e1575gf*, promotes longevity. A *tra‐3(lf)* mutation was used to maintain *tra‐1(e1575gf)* mutant animals that are essentially females. (f‐g') Both mutational inactivation (f and f') and RNA interference‐mediated depletion (g, g') of *daf‐16* suppress lifespan extension in *fem‐3(‐)* mutant (*i.e., tra‐1* hyperactive) animals. (h‐i') Mutational inactivation (h, h') and depletion (i, i') of *daf‐16* suppress the longer lifespan of *tra‐1(e1575gf)* mutants. On panels (b, c, and e‐i), Kaplan–Meier lifespan curves (log‐rank tests) while on panels (b', c', e'‐i'), the corresponding mean survival data (independent samples *t* tests with Bonferroni correction) are shown. On the latter, *p < .05, **p < .001, ***p < .0001; NS: not significant. On panels (a'‐i'), bars represent ±*SEM*. Statistics and data are included in Table S1. On panels (g, g' and i, i'), “ev” denotes empty vector (animals were fed with bacteria expressing the empty vector only). *tra‐3(lf)* indicates the loss‐of‐function allele *tra‐3(e1767)*. On panels (a and d), arrows indicate activations, bars represent inhibitory regulatory interactions. On panels (e‐g'), wild‐type corresponds to wild‐type hermaphrodites. On panels (a, c and e‐i), animals were maintained at 25°C

We measured the lifespan of nematodes that were maintained in groups: 60–70 hermaphrodites and five males were placed on each test plate (this sex ratio approaches to those found in natural *C. elegans* populations in which males are present at low frequency; Brenner, 1974). In good accordance with previous data (Johnson & Wood, 1982; Johnson & Hutchinson, 1993), under these circumstances―such population density and sex ratio enable both individual and social behavioral patterns including response to crowding/dauer pheromone, mating, and being in hiding―hermaphrodites lived about 2 days longer than males at 25°C (Figure 1a,a'). This is a nearly 20% longevity difference in favor of hermaphrodites. Similar results were obtained when animals were grown on media lacking the DNA synthesis inhibitor FUdR (5‐fluoro‐2‐deoxyuridine; Figure S1), which is generally used in *C. elegans* lifespan assays to confer sterility to the treated animals and known to affect longevity in the wild‐type at higher temperatures (Angeli et al., 2013). The tendency of hermaphrodites to live longer than males was also evident when populations were maintained at 20°C (Figure S2). Thus, the longer lifespan of hermaphrodites over males in mixed populations with hermaphrodite abundance appears to be established largely independently of several environmental conditions, raising the potential involvement of genetic (regulatory) factors in determining sex differences in lifespan.

### 2.2 In nematodes defective for insulin/IGF‐1 signaling, the longevity advantage of hermaphrodites over males depends on DAF‐16/FOXO activity

Next, we aimed to explore the regulatory mechanisms underlying the hermaphrodite bias in longevity. The IIS pathway plays a pivotal role in the control of *C. elegans* aging (Kenyon, 2010; Lin, Dorman, Rodan & Kenyon, 1997; Ogg et al., 1997). Upon ligand binding, the insulin/IGF‐1 plasma membrane receptor DAF‐2 (constitutive dauer formation) activates a cascade of downstream cytoplasmic kinases, which eventually inhibits the FOXO‐like transcription factor DAF‐16 (Ogg et al., 1997; Figure 1B). When IIS is lowered, DAF‐16 effectively translocates into the nucleus to dictate the expression of target genes required for lifespan extension, stress resistance, and dauer larval formation (dauer is an alternative, nonaging developmental diapause triggered by starvation, crowding, and high temperatures in the wild‐type; Fielenbach & Antebi, 2008; Vellai et al., 2003). We found that in long‐lived *daf‐2(‐)* loss‐of‐function mutant strains maintained at temperatures permitting reproductive growth, hermaphrodites also tend to live longer than males (Figure 1c,c' and Figure S3). Note that a previous study reported a male longevity advantage when animals defective for DAF‐2 were fed killed *E. coli* bacteria as a food, showing that males are more susceptible to live *E. coli* toxicity (Gems & Riddle, 2000). *daf‐16* deficiency however suppressed the longer lifespan hermaphrodites exhibit over males in *daf‐2(‐)* mutant genetic backgrounds (Figure 1c,c' and Figure S3). These data indicate that DAF‐16 mediates increased hermaphrodite longevity in this sensitized IIS‐defective genetic background (in well‐fed wild‐type animals, *daf‐16* is largely repressed by IIS).

### 2.3 The longevity advantage of hermaphrodites over males depends on TRA‐1 activity

How is *daf‐16* controlled differently in hermaphrodites and males? To test whether the nematode sex‐determination cascade (Figure 1d) is implicated in the sex‐specific regulation of aging, we monitored the lifespan of mutant animals with decreased or elevated TRA‐1 activity. According to these results, *tra‐1(‐)* mutations transforming animals with XX (hermaphrodite) karyotype into males significantly reduced lifespan (Figure 1e,e'). Consistent with this finding, a *tra‐1* gain‐of‐function (gf) mutation, *e1575*, increased lifespan by approximately 30% (animals with hyperactive TRA‐1 function are feminized; Figure 1e,e'). Animals bearing the *tra‐1* gf allele *e1575* were propagated in a *tra‐3(‐)* mutant background, which itself significantly limited lifespan. Furthermore, *fem‐3(‐)* (feminization of hermaphrodite and male animals) mutants similarly hyperactive for TRA‐1 function (Figure 1d) likewise lived 2–3 days longer than controls (Figure 1e,e'). We found that inactivation of DAF‐16 completely eliminates lifespan extension in *tra‐1(gf)* and *fem‐3(‐)* mutant animals (Figure 1f‐i'). Double‐mutant animals that are defective for DAF‐16 activity and hyperactive for TRA‐1 function lived nearly as short as *daf‐16* single mutants (Figure 1f‐i'). We conclude that TRA‐1 promotes longevity in hermaphrodites by enhancing *daf‐16* activity. In other words, TRA‐1 strengthens the function of *daf‐16* in aging control, explaining why hermaphrodites live significantly longer than males in populations containing both sexes. At first sight, these results were somewhat unexpected as TRA‐1 had previously been known as a transcriptional repressor rather than an activator (Berkseth et al., 2013; Chen & Ellis, 2000; Conradt & Horvitz, 1999; Hargitai et al., 2009; Mason et al., 2008; Schwartz & Horvitz, 2007; Szabó et al., 2009; Yi et al., 2000). In case of mammalian GLI proteins, however, both activation and inhibition functions were observed (Hui & Angers, 2011).

### 2.4 The expression of *daf‐16* is influenced by TRA‐1

**Figure 2:**
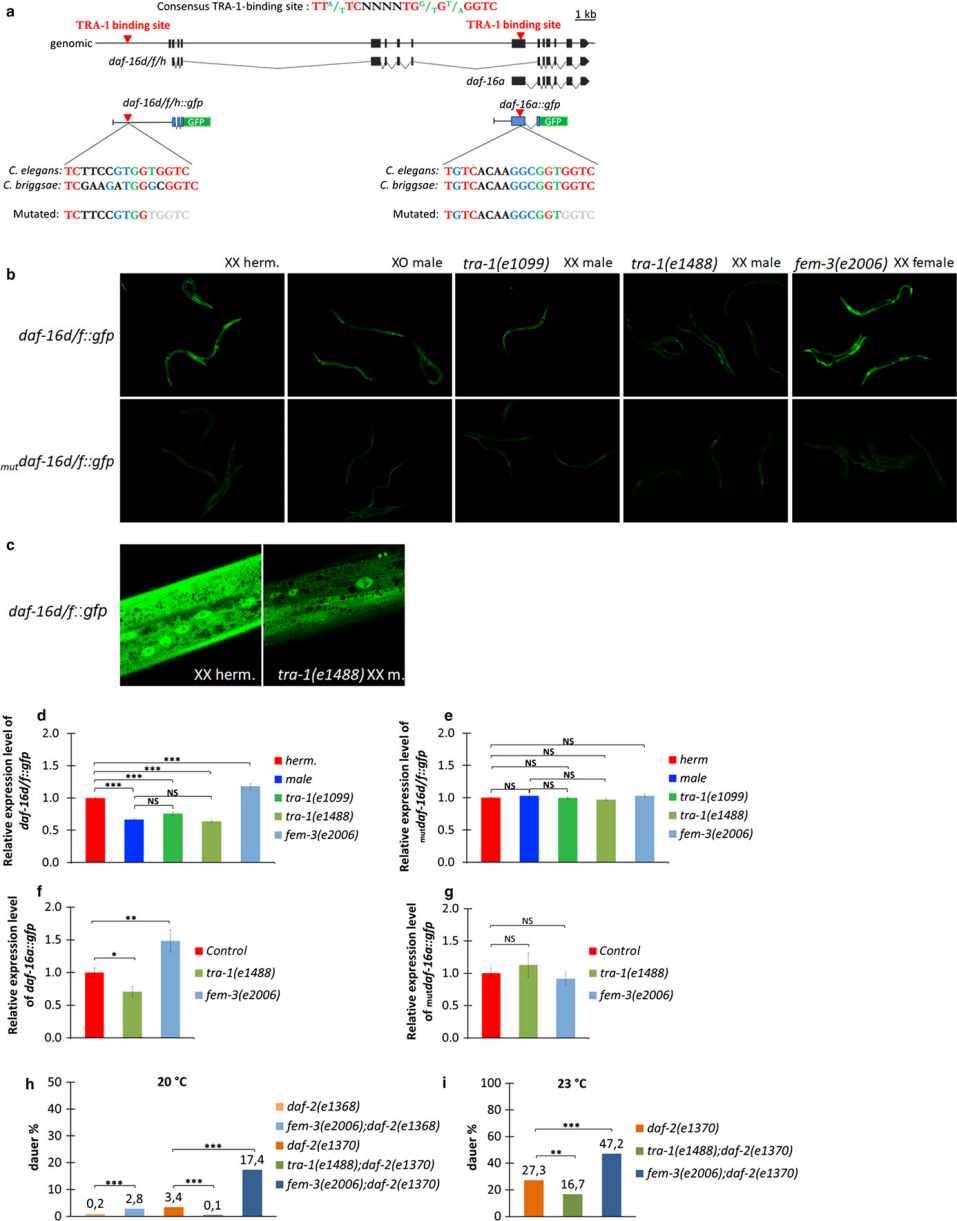
TRA‐1 promotes *daf‐16* expression in hermaphrodites. (a) Structure of two *daf‐16* isoforms, *d/f* and *a*. Black boxes correspond to exonic sequences, connecting lines represent introns. Red triangles indicate conserved TRA‐1‐binding sites (the consensus sequence is shown on the top; red letters indicate identical nucleotides; green nucleotides indicate conserved nucleotides; N denotes arbitrary nucleotides). The structures of *daf‐16d/f::gfp* and *daf‐16a::gfp* reporters are also shown. Blue letters indicate difference from the consensus sequence, and gray letters indicate nucleotides that are missing in the mutated constructs. (b) *daf‐16d/f::gfp* expression is decreased in XO males and in *tra‐1(‐)* mutant XX males and increased in *fem‐3(‐)* mutant backgrounds (top row). _*mut*_ *daf‐16d/f::gfp* (a mutated derivative of the wild‐type construct, lacking several nucleotides in the predicted TRA‐1 binding site) expression is independent of *fem‐3* and *tra‐1* activities (bottom row). All fluorescence pictures were taken with the same exposure time (500 ms) and magnification (100×). (c) High‐resolution confocal microscopy images of *daf‐16d/f* expression in a wild‐type (control, left) vs. a *tra‐1(‐)* mutant (right) animal. Fluorescence pictures were taken with the same exposure setting. (d, e) Quantification of the relative expression intensity of *daf‐16d/f::gfp* (*d*) and _*mut*_ *daf‐16d/f::gfp* (*e*) reporters. *daf‐16d/f::gfp* expression is increased in *fem‐3(‐)* mutant animals but decreased in XO males and in *tra‐1(‐)* mutant XX animals. Interestingly, the hypomorphic *tra‐1(e1488)* mutation has a stronger effect on reporter expression than the genetic null allele *tra‐1(e1099)* does which may be due to maternal effect (*tra‐1* null mutants can be maintained as heterozygous animals) (d). In contrast, expression levels of _*mut*_ *daf‐16d/f::gfp* is largely independent of TRA‐1 activity (e). (f, g) Expression intensity of *daf‐16a::gfp* (*f*) and _*mut*_ *daf‐16a::gfp* (*g*) reporters at the late L1 larval stage when dauer development is initiated. Expression of _*mut*_ *daf‐16a* appears to be independent of TRA‐1. In panels (d‐g), *p < .05; **p < .01, ***p < .001, independent samples *t* tests; bars represent ±*SEM*; “NS” denotes not significant. Statistics and data are included in Table S2. (h, i) Dauer development in *daf‐2(‐)* mutants is increased by FEM‐3 deficiency and decreased by TRA‐1 deficiency. The percentage of dauer larvae is determined at 20°C (h) and 23°C (i). **p < .01, ***p < .001; chi‐squared tests with Bonferroni correction. Statistics and data are included in Table S3

*daf‐16* encodes several isoforms (Figure 2a), three of which (*R13H8.1b, d,* and *f*, according to the current version [WA261] of WormBase: http://www.wormbase.org/) are known to influence the rate of the aging process (Kwon, Narasimhan, Yen & Tissenbaum, 2010). Performing a sequence analysis, we have identified two conserved TRA‐1‐binding sites (Conradt & Horvitz, 1999) in the *daf‐16* locus. The sites were also found in the orthologous genomic regions of *C. briggsae*, a closely related *Caenorhabditis* species (Figure 2a). These data, together with results provided by a previous chromatin immunoprecipitation assay followed by deep sequencing (ChIP‐seq; Berkseth et al., 2013), raised the possibility of a direct regulatory interaction between TRA‐1 and *daf‐16* isoforms involved in aging control. One of these consensus TRA‐1‐binding sites is located at 3‐kilobase (kb) upstream of the *daf‐16d/f* isoforms, while the other is located within the first exon of the *daf‐16a* isoform (exonic sequences often serve as binding elements for transcriptions factors; Stergachis et al., 2013). The ChIP‐seq analysis provided by Berkseth and colleagues also identified two potential TRA‐1‐binding sites in the *daf‐16* coding region (Berkseth et al., 2013) which are however slightly diverged from the canonical one (Conradt & Horvitz, 1999; Hargitai et al., 2009) and the *daf‐16*‐specific TRA‐1‐binding sites identified by our present study (Figure 2a). To assess the functionality of these potential TRA‐1‐binding sites, we generated two transgenic strains expressing isoform‐specific *gfp*‐ (green fluorescent protein) tagged *daf‐16* reporter constructs, *daf‐16d/f::gfp* and *daf‐16a::gfp* (Figure 2a). Both constructs include the TRA‐1‐binding site identified in the corresponding regulatory region. At the late L4 larval and young adult stages when IIS begins to affect lifespan, *daf‐16d/f::gfp* was expressed in almost all somatic cells of both sexes, mainly in the cytoplasm, but nuclear presence was also detectable, and at significantly higher levels in XX hermaphrodites than in XO males (Figure 2b‐d). Animals transgenic for a mutated version of the reporter, _*mut*_ *daf‐16d/f::gfp*, lacking five critical bases in the predicted TRA‐1‐binding sequence (gray letters in Figure 2a) displayed decreased expression when compared to *daf‐16d/f::gfp* expression levels at the same stages and exhibited no response in expression to the sex and TRA‐1 activity (Figure 2b,e). To further confirm that TRA‐1 promotes rather than represses *daf‐16d/f* expression, we examined reporter activity in *fem‐3(‐)* and *tra‐1(‐)* mutant backgrounds. Indeed, *daf‐16d/f::gfp* was strongly upregulated in *fem‐3(‐)* and downregulated in *tra‐1(‐)* mutant animals at the L4 larval/young adult stages (Figure 2b‐d). In contrast, mutational inactivation of *fem‐3* or *tra‐1* was not able to alter the expression of the mutated _*mut*_ *daf‐16d/f::gfp* reporter (Figure 2b,e). Confocal microscopy also demonstrated that intracellular levels of *daf‐16d/f::gfp* expression are markedly attenuated by TRA‐1 deficiency (Figure 2c). Sex differences in *daf‐16d/f::gfp* expression remained on in later developmental stages and throughout adulthood (Figures S4 and S5, and Tables S6 and S7). Together, we suggest that TRA‐1 enhances the transcriptional activity of *daf‐16d/f* presumably through the predicted binding site.

*daf‐16a::gfp* was mainly expressed in neuronal and hypodermal cells in the head and tail body regions (Figure S6). Its expression intensity was significantly decreased in *tra‐1(‐)*, but enhanced in *fem‐3(‐)*, mutant worms at the L1/2 larval stages when developmental decision between normal reproductive growth and dauer larva formation occurs (Figure 2f and Figure S6). A binding site mutant version of the reporter, _*mut*_ *daf‐16a::gfp* (Figure 2a), also displayed decreased expression levels, as compared with the corresponding wild‐type reporter (Figure 2g and Figure S6). Moreover, at these larval stages, _*mut*_ *daf‐16a::gfp* expression appeared to be largely independent of TRA‐1 activity. Thus, the expression of *daf‐16a* is also enhanced by TRA‐1. Interestingly, after the L1/2 larval stages when the reproductive growth vs. dauer development decision is already determined, *daf‐16a::gfp* expression appeared to be no longer activated by TRA‐1 (Figure S7 and Table S8). At the L4 larval and young/aged adult stages, *daf‐16a* was expressed at similar or even higher levels in XO males and *tra‐1(‐)* mutant XX animals than in XX hermaphrodites.

Thermosensitive (ts) *daf‐2(‐)* ^*ts*^ mutant animals enter into the dauer larval stage at the restrictive temperature (25°C). The manifestation of the Daf‐constitutive phenotype in *daf‐2(‐)* ^*ts*^ mutants requires DAF‐16 activity; *daf‐2(‐)* ^*ts*^ *; daf‐16(‐)* double‐mutant animals grow as reproductive adults even at 25°C. *daf‐16a* isoform is known to promote dauer development but has no effect on lifespan (Kwon et al., 2010). We found that dauer larval formation in *daf‐2(‐)* ^*ts*^ mutants is increased in *fem‐3(‐)* and inhibited in *tra‐1(‐)* mutant backgrounds at temperatures (20–23°C) where only a portion of the population develops as dauer larvae (Figure 2h,i). These data imply that TRA‐1 also modulates IIS by enhancing *daf‐16a* activity in controlling the decision between reproductive growth and dauer larval development in hermaphrodites.

### 2.5 A functional *daf‐16d/f* transgene promotes longevity more effectively and is expressed at higher levels in hermaphrodites than in males

**Figure 3:**
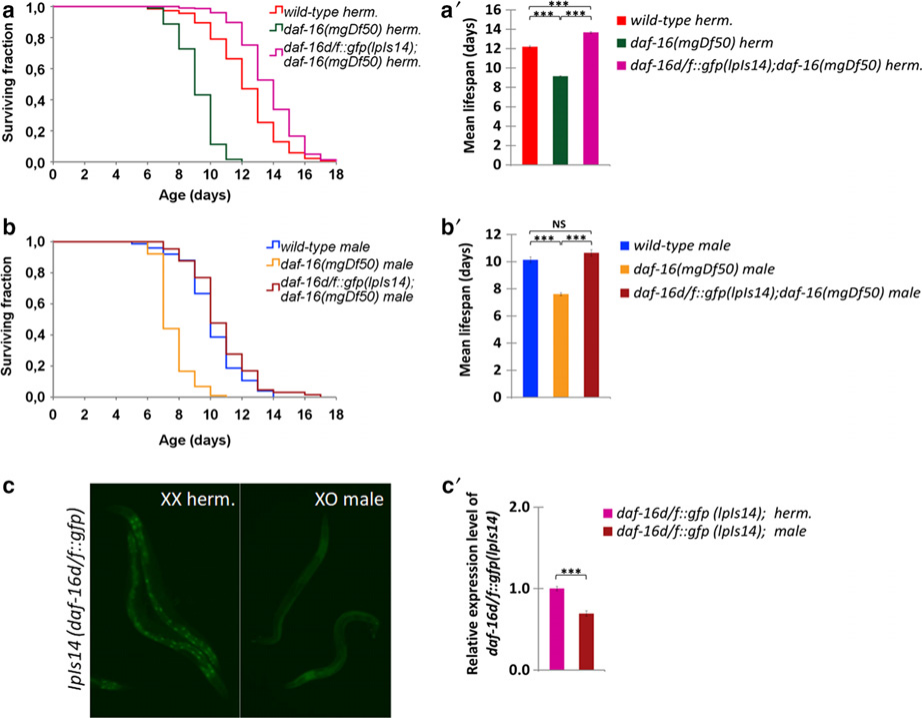
A functional, full‐length *daf‐16d/f::gfp* transgene (*lpIs14*) extends lifespan more effectively in hermaphrodites than in males. (a) Lifespan curve of wild‐type hermaphrodites, *daf‐16(‐)* mutant hermaphrodites, and *daf‐16(‐)* mutant hermaphrodites transgenic for *lpIs14*. *lpIs14* denotes an integrated, full‐length *daf‐16d/f::gfp* transgene (Kwon et al., 2010). It rescues normal lifespan in animals defective for DAF‐16. (a') The corresponding mean lifespan data. (b) Lifespan curve of wild‐type males, *daf‐16(‐)* mutant males and *daf‐16(‐)* mutant males transgenic for *lpIs14*. (b') The mean lifespan data. *lpIs14* promotes longevity more significantly in *daf‐16(‐)* mutant hermaphrodites (a, a') than in *daf‐16(‐)* mutant males (b, b'). In panels (a' and b'), NS indicates not significant, *** indicates *p* < .001, log‐rank and independent samples *t* test. (c) Expression of *lpIs14* in young hermaphrodites and males. (c') Relative expression levels of *lpIs14* in hermaphrodites vs. males (the expression is higher in the former). Bars represent ±*SEM*, *** indicates *p* < .001, independent samples *t* test. For statistics and data, see Tables S1 and S2

*daf‐16* encodes several isoforms, among which *daf‐16d/f*, together with *daf‐16b*, were identified as the main transcripts that regulate nematode lifespan (Kwon et al., 2010). We crossed an integrated, full‐length (translational fusion) *daf‐16d/f::gfp* reporter transgene, *lpIs14* (Kwon et al., 2010), into a *daf‐16(‐)* mutant background, and found that it is capable of rescuing normal lifespan in both hermaphrodites and males (Figure 3a‐b'). However, the lifespan extending effect of *lpIs14* transgene was more evident in hermaphrodite animals than in males. Under conditions of 10:1 hermaphrodite:male population ratio, *daf‐16(‐)* mutant hermaphrodites transgenic for *lpIs14* lived even longer than wild‐type hermaphrodites (*p* < .0001; see Figure 3a,a'), whereas the lifespan of *daf‐16(‐); daf‐16d/f* (*lpIs14*) males did not exceed that of the wild‐type (Figure 3b,b'). When males were maintained in single‐sex groups, *daf‐16(‐)* mutants transgenic for *Ipls14* lived only a slightly longer than wild‐type males (*p* < .05; Figure S8). Consistent with these data, *lpIs14* was expressed at higher levels in hermaphrodites, as compared with males, in an otherwise wild‐type background (Figure 3c,c' and Table S2). Interestingly, the expression was also obvious in the nucleus of intestinal cells in hermaphrodites but not in males. Hence, the expression of *daf‐16d/f*, two *daf‐16* isoforms that control the rate at which cells age, is influenced by the sex of the animal in favor of hermaphrodites. This can explain why hermaphrodites live longer than males in populations containing both genders, and with hermaphrodite excess.

### 2.6 TRA‐1 binds to the two novel regulatory sequences in the *daf‐16* locus

**Figure 4:**
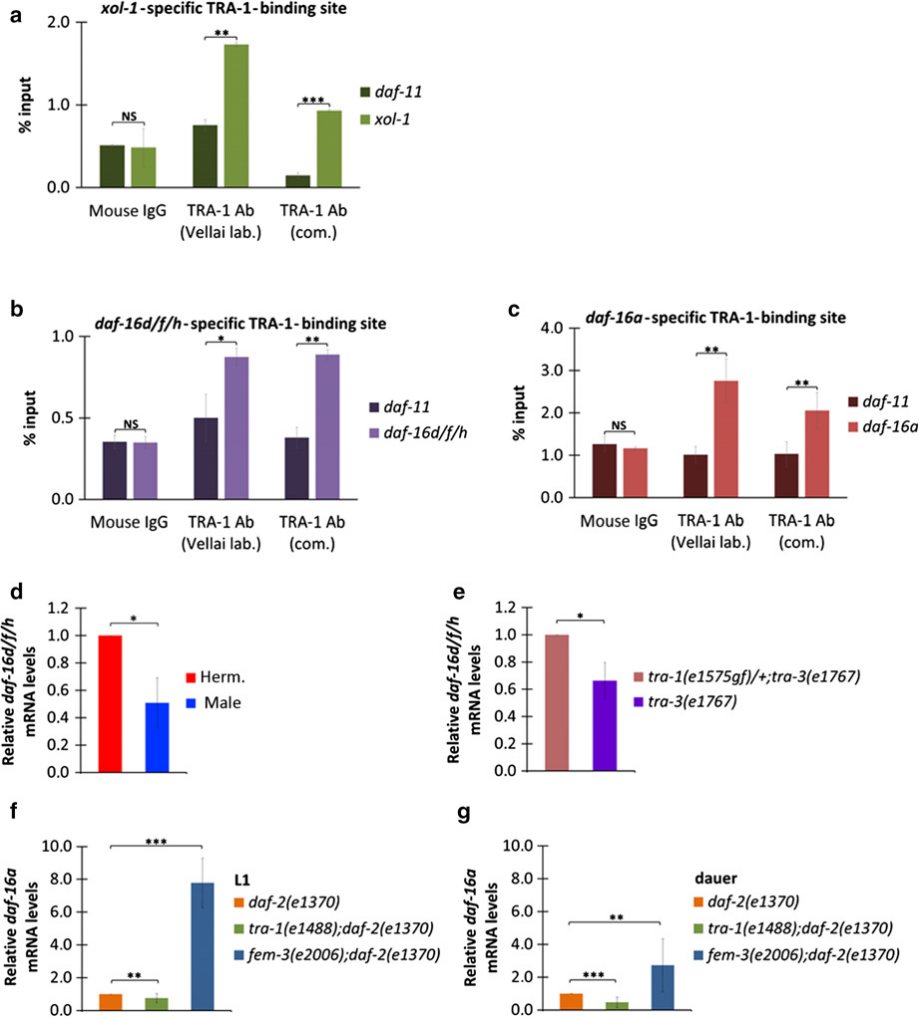
The *daf‐16* isoforms *d/f* and *a* are direct targets of the sex‐determining factor TRA‐1. (a‐c) ChIP (chromatin immunoprecipitation) data showing TRA‐1 binding to target sequences. (a) TRA‐1 binds to a *xol‐1*‐specific genomic fragment containing the conserved binding site (positive control), but not to *daf‐11*‐specific one (inner negative control). (b) TRA‐1 binds to a *daf‐16d/f* isoform‐specific DNA fragment that contains the binding sequence (Figure 2a) in vivo. (c) TRA‐1 binds to a *daf‐16a*‐specific genomic fragment with the conserved binding site (Figure 2a). On panels (a‐c), “Vellai lab”: TRA‐1 antibody generated by our laboratory (see Figures S9 and S10); “com”: commercially available TRA‐1 antibody; Ab: antibody; mouse IgG: negative control. Bars represent ±*SEM* *p < .05; **p < .01; ***p < .001. Independent samples *t* tests with Bonferroni correction. Statistics and data are included in Table S4. (d) *daf‐16d/f* transcript levels are significantly higher in wild‐type hermaphrodites than in males. (e) *tra‐1(gf)* mutation increases *daf‐16d/f* transcript levels, as compared with controls. (f, g) At the late L1 (f) and dauer (g) larval stages, the expression of *daf‐16a* is decreased in *tra‐1(‐)* but increased in *fem‐3(‐)* mutant backgrounds. In panels (d‐g), quantitative RT–PCR data are shown; bars represent mean ± *SD*, *p < .05, **p < .01; ***p < .001, Pair Wise Fixed Reallocation Randomization test. Statistics and data are included in Table S5

To provide evidence for a direct regulatory interaction between the transcription factor TRA‐1 and its potential target gene *daf‐16*, we generated a TRA‐1‐specific antibody (Figures S9 and S10) and obtained a commercially available TRA‐1‐specific antibody (see the Materials and Methods section) to be used for a chromatin immunoprecipitation (ChIP) assay (Figure 4a‐c). In these experiments, TRA‐1 was able to bind to a genomic fragment containing the canonical TRA‐1‐binding site identified at 3 kb upstream of the *daf‐16d/f* coding region (Figure 4b). Binding of TRA‐1 to the regulatory region of *daf‐16d/f* was as effective as to the regulatory region of *xol‐1* (positive control), a known TRA‐1 target gene (Figure 4a,b; Hargitai et al., 2009). In contrast, TRA‐1 did not bind to a genomic fragment from *daf‐11* locus (negative control). Positive results were also obtained when monitoring the *daf‐16a*‐specific binding site (Figure 4c). These ChIP data were further supported by a set of quantitative real‐time PCR experiments. *daf‐16d/f* transcript levels were measured in hermaphrodites vs. males, and in wild‐type vs. TRA‐1 hyperactive genetic backgrounds. We found that expression levels of *daf‐16d/f* are higher in hermaphrodites than in males (Figure 4d), and increased in *tra‐1(gf)* mutant animals, relative to control (Figure 4e). Similarly, *daf‐16a* transcript levels were decreased in *tra‐1(‐)* and elevated in *fem‐3(‐)* mutant backgrounds at the late L1 larval stage when *daf‐16a* may exert its effect on initiating dauer larval development (Figure 4f), and also in dauer larvae (Figure 4g). Together, it can be established that TRA‐1 directly promotes the expression of *daf‐16d/f* and *a* isoforms.

An unbiased TRA‐1‐specific ChIP‐seq study performed at four different developmental time points also identified TRA‐1 binding to *daf‐16* (Berkseth et al., 2013), but the binding sites the authors determined in the *daf‐16* locus are not the same as those we identified in this study. Here, we carried out ChIP‐qPCR (more sensitive and powerful than ChIP‐seq) on ultrasound fragmented chromatin to 500‐bp‐long fragments, prepared from mixed‐stage animals. We used a positive (*xol‐1*) and a negative (*daf‐11*) control regions, two different TRA‐1‐specific antibodies, and IgG as a negative control (Figure 4). In the ChIP‐seq analysis, 184 TRA‐1‐binding sites were identified, which are fewer than typical for site‐specific transcription factors. Indeed, several previously described TRA‐1 target genes such as *egl‐1*,* ceh‐30,* and *lin‐39* (Conradt & Horvitz, 1999; Schwartz & Horvitz, 2007; Szabó et al., 2009) remained unidentified by this ChIP‐seq analysis (Berkseth et al., 2013). However, we know that a direct evidence for the functionality of the TRA‐1‐binding site we determined in the *daf‐16* locus would be the elimination of the binding site by CRISPR/Cas technology which would block the regulatory interaction between TRA‐1 and *daf‐16d/f* and *a* isoforms in vivo.

## 3 DISCUSSION

**Figure 5:**
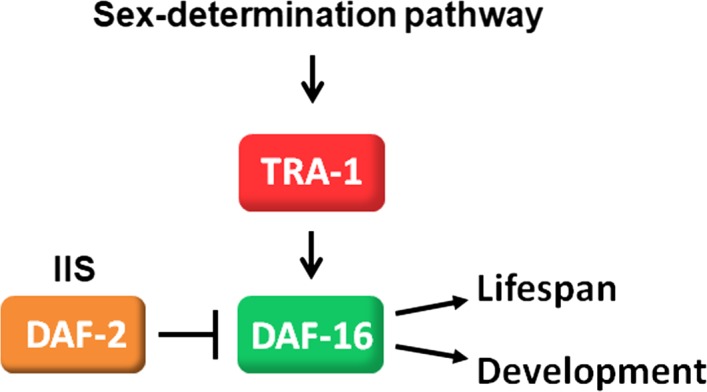
Model for the sex‐specific regulation of aging in *C. elegans*. TRA‐1, the terminal transcription factor of the nematode sex‐determination pathway, promotes the expression of *daf‐16* (*d/f* and *a* isoforms) to extend lifespan and to promote dauer development in hermaphrodites. As TRA‐1 is essentially active only in hermaphrodites, hermaphrodites live significantly longer than males. “IIS” denotes insulin/IGF‐1 signaling. Arrows indicate activations, and the bar represents inhibitory regulatory interaction

*Caenorhabditis elegans* is a tractable model system to study the molecular mechanisms underlying sex‐specific differences in various biological processes and anatomical features. In this work, we explored a novel regulatory interaction that determines lifespan and reproductive growth unequally between hermaphrodite and male animals. First, we observed that both wild‐type and IIS‐deficient *daf‐2(‐)* mutant hermaphrodites live significantly longer than the corresponding males (Figure 1 and Figures S1–S3). Sex differences in longevity in *daf‐2(‐)* mutants disappeared in *daf‐16(‐)* mutant genetic backgrounds (Figure 1 and Figure S3). Thus, the sex‐specific regulation of nematode lifespan depends on DAF‐16 activity. Next, we showed that the master sex‐determining factor TRA‐1 promotes the transcriptional activity of certain *daf‐16* isoforms, *d/f* and *a*. TRA‐1 and these *daf‐16* isoforms hence act in the same genetic pathway to modulate lifespan or development. As DAF‐16 functions as the main target of IIS in the regulation of lifespan and development, TRA‐1, and thereby the sex‐determination machinery, is an important modulator of this signaling system (Figure 5). This implies that IIS is adjusted in a sex‐specific way, leading to significant sex differences in the activity of several biological processes. Indeed, the expression of *daf‐16d/f* playing an important role in longevity control (Kwon et al., 2010) is elevated by TRA‐1 in hermaphrodites but not in males (Figures 2, 3, 4). Depending on population density and the ambient temperature, *daf‐16a* controls the decision between reproductive growth and dauer larva development (Figures 2 and 4). TRA‐1 also increases the expression of this *daf‐16* isoform in hermaphrodite animals. These regulatory interactions elucidate the hermaphrodite bias toward a longer lifespan (this study) and increased dauer larval formation (Vellai, McCulloch, Gems & Kovács, 2006). Similarly, a marked sex‐specific difference was previously observed in *C. elegans* learning capacity, a trait that also relies on IIS (Vellai et al., 2006). It would be relevant to examine whether the TRA‐1–*daf‐16* regulatory axis is involved in the control of associative learning. In case of positive results, one could provide an explanation for the tendency of hermaphrodites to perform an associative learning paradigm more effectively. In nematodes, lipid metabolism and stress resistance are also influenced by DAF‐2 and DAF‐16 (Ashrafi et al., 2003; Scott, Avidan & Crowder, 2002). Through enhancing the activity of certain *daf‐16* isoforms, TRA‐1 may also strengthen these biological processes in hermaphrodite animals.

Until recently, DAF‐16/FOXO was known to be regulated predominantly at posttranscriptional levels (via phosphorylation by the serine‐threonine kinase Akt; Figure 1b; Murphy & Hu, 2013). TRA‐1 however influenced the transcription of certain *daf‐16* isoforms, *d/f* and *a* (Figure 2 and 4). This raises the relevant question of whether increasing merely *daf‐16* transcription is sufficient for lifespan extension. In animals transgenic for a functional *daf‐16* reporter construct, *lpIs14* (Kwon et al., 2010), lifespan was shown to increase proportionately with the copy number of the transgene (Bansal et al., 2014). Furthermore, the chromatin remodeler SWI/SNF complex was demonstrated to extend lifespan through controlling *daf‐16* transcription. These data revealed that elevating *daf‐16* transcript levels is indeed capable of extending lifespan significantly.

In the nematode sex‐determination cascade, FEM‐1 inhibits TRA‐1 (Figure 1d), which is orthologous to human GLI proteins (Robbins, Fei & Riobo, 2012; Zarkower & Hodgkin, 1992) acting as downstream effectors of Hh signaling. Accumulating evidence indicates that Hh signaling plays a pivotal role in mammalian sexual differentiation (Franco & Yao, 2012) and that the mammalian Fem1b protein, an orthologue of FEM‐1, also suppresses the transcriptional activity of GLI1 (Gilder, Chen, Jackson, Jiang & Maher, 2013). In addition, Hh signaling regulates whole‐body energy metabolism via activating the Akt/FOXO pathway (Zhang, Cheng, Wang, Leung & Mak, 2017) and also influences IIS activity in certain developmental events (Lipinski et al., 2005). This is particularly interesting as in mammals, IIS is implicated in the sex‐specific regulation of tissue differentiation (Lam, Shah & Brosens, 2012; Pitetti et al., 2013). These data prompted us to perform an in silico analysis of the human genome for the presence of conserved GLI binding sites in the *FOXO3* locus (Figures S11 and S12). In mammals, FOXO proteins are encoded by four genes, *FOXO1*,* 3*,* 4,* and *6*, and allelic variations of *FOXO3* have been correlated with longevity in numerous human populations (Martins, Lithgow & Link, 2016). By performing a locus‐specific sequence analysis, we uncovered conserved GLI binding sites in the regulatory regions of human *FOXO* genes, especially *FOXO3* (Figures S11 and S12). A potential consensus site found in the first intronic sequence of *FOXO3* showed a strong conservation across the orthologous regions of mammalian, in particular primate, genomes. In addition, accumulating evidence indicates that Hh signaling plays a fundamental role in mammalian sexual differentiation (Franco & Yao, 2012; Wang et al., 2013). Based on this evolutionary conservation and gender‐specific activity of Hh signaling, we speculate that a similar regulatory interaction between Hh signaling and FOXO‐like transcription factors may also operate in humans to determine lifespan unequally in the two genders. Although nematodes lack certain components of the canonical Hh signaling pathway and primary sex‐determination in mammals is strictly chromosomal (*i.e.,* depends on the presence of chromosome Y), genetic interaction between functionally conserved proteins and genes (TRA‐1/GLI and *daf‐16/FOXO3*) may affect lifespan in a sex‐specific manner across divergent animal taxa. Together, these data raise the possibility that molecular interactions between TRA‐1/GLI and DAF‐16/FOXO proteins are evolutionarily conserved from worms to humans. It is possible that in mammals, the presence of chromosome Y somehow determines the activity of Hh signaling in a sex‐specific manner, which in turn influences IIS through a GLI–FOXO regulatory interaction.

In mammals, IIS controls various cellular, physiological, and developmental functions, including apoptosis, aging, metabolism, systemic body growth, self‐renewal of stem cells, and behavior. Many of such functions manifest in a sex‐specific manner. For example, in humans, the control of behavior, sensory information transmission, learning, and memory processing all depend on IIS and display a marked sex bias: women tend to behave less aggressively, have a better sense of smell, and learn skills faster than men (Londorsf, Eberly & Pusey, 2004). Uncovering the regulatory role of the *C. elegans* sex‐determining protein TRA‐1 in *daf‐16* activity may help to understand better how IIS affects diverse biological processes unequally between women and men.

## 4 EXPERIMENTAL PROCEDURES

### 4.1 Strains and genetics

Nematodes were maintained and propagated on Nematode Growth Medium‐ (NGM) containing plates and fed with *Escherichia coli OP50* bacteria. The following *C. elegans* strains were used in this study: Bristol (N2) as wild‐type.

TTV395 *tra‐1(e1099)/hT2qIs48(I)III;*
CB2823 *tra‐1(e1488)III/eDp6(III;f);*
CB3769 *tra‐1(e1575)/+III; tra‐3(e1767)IV;*
CB3844 *fem‐3(e2006)IV;*
CB1370 *daf‐2(e1370)III;*
DR1572 *daf‐2(e1368)III;*
CF1038 *daf‐16(mu86)I;*
GR1307 *daf‐16(mgDf50)I;*
TTV522 *daf‐2(e1370)III; daf‐16(mu86)I;*
TTV600 *tra‐1(e1488)/+, daf‐2(e1370)III;*
TTV344 *tra‐1(e1488)/+, daf‐2(e1368)III;*
TTV309 *fem‐3(e2006)IV; daf‐2(e1370)III;*
TTV323 *fem‐3(e2006)IV; daf‐2(e1368)III*;
TTV338 *fem‐3(e2006)IV; daf‐16(mu86)I*;
TTV337 *tra‐1(e1575)/+III; tra‐3(e1767)IV; daf‐16(mu86)I*;
TTV310 *unc‐119(ed3)III; eluIs300[Pdaf‐16d/f::gfp+unc‐119(+)]*;
TTV336 *unc‐119(ed3)III; eluIs302[P∆daf‐16d/f::gfp+unc‐119(+)]*;
TTV421 *unc‐119(ed3)III; eluEx370[Pdaf‐16a::gfp+unc‐119(+)]*;
TTV432 *unc‐119(ed3)III; eluEx374[P∆daf‐16a+unc‐119(+)]*;
TTV318 *tra‐1(e1488)/+III; unc‐119(ed3)III; eluIs300[Pdaf‐16d/f::gfp+unc‐119(+)]*;
TTV341 *tra‐1(e1099)/hT2qIs48(I)III; unc‐119(ed3)III; eluIs300[Pdaf‐16d/f::gfp+unc‐119(+)]*;
TTV347 *tra‐1(e1575)/+III; unc‐119(ed3)III; tra‐3(e1767)IV; eluIs300[Pdaf‐16d/f::gfp+unc‐119(+)]*;
TTV340 *fem‐3(e2006)IV; unc‐119(ed3)III; eluIs300[Pdaf‐16d/f::gfp+unc‐119(+)]*;
TTV329 *him‐5(e1490)V; unc‐119(ed3)III; eluIs300[Pdaf‐16d/f::gfp+unc‐119(+)]*;
TTV321 *tra‐1(e1488)/unc‐119(ed3)III; eluIs302[P∆daf‐16d/f::gfp+unc‐119(+)]*;
TTV469 *tra‐1(e1099)/unc‐119(ed3)III; eluIs302[P∆daf‐16d/f::gfp+unc‐119(+)]*;
TTV327 *fem‐3(e2006)IV; unc‐119(ed3)III; eluIs302[P∆daf‐16d/f::gfp+unc‐119(+)]*;
TTV428 *fem‐3(e2006)IV; unc‐119(ed3)II; eluEx370[Pdaf‐16a::gfp+unc‐119(+)]*;
TTV472 *tra‐1(e1099)/hT2qIs48(I)III; eluEx370[Pdaf‐16a::gfp+unc‐119(+)]*;
TTV433 *fem‐3(e2006)IV; unc‐119(ed3)III; eluEX374[P∆daf‐16a+unc‐119(+)]*;
TTV427 *tra‐1(e1099)/+ III; unc‐119(ed3)III; eluEX374[P∆daf‐16a+unc‐119(+)];*
HT1889 *daf‐16(mgDf50)I; unc‐119(ed3)III; lpIs14*.

### 4.2 Lifespan assays

Lifespan assays were carried out at 25°C. *daf‐2(‐)* ^*ts*^ mutant animals were maintained at 20°C until the L4 larval stage, then transferred at 25°C (otherwise indicated), and scored for mean lifespan. For synchronization, 20–30 gravid, well‐fed adults were transferred to a new agar plate containing NGM seeded with *E. coli OP50* bacteria to lay embryos for 4–5 hr and then removed. Alternatively, embryos were prepared by NaOH–hypochlorite treatment. Approximately 60–70 F1 young (nongravid) adults were transferred to new NGM plates supplemented with 300–400 mg/ml FUdR (5‐fluoro‐2′‐deoxyuridine, Sigma; *t* = 0). Sterile F1 adults were then assayed. Animals that climbed up the wall of plastic dishes or exhibited a protruded vulva phenotype were excluded from the analysis. Animals were considered dead when they stopped pharyngeal pumping and responding to touching. In hermaphrodite vs. male lifespan assays, approximately 50–60 hermaphrodites and five to six males were maintained on each plate. SPSS 17 software was used to calculate mean lifespan and perform statistical analysis. *p* values for comparing Kaplan–Meier survival curves between two groups were determined using log‐rank (Mantel–Cox) tests, and *p* values for comparing mean lifespans were determined using independent samples *t* tests with Bonferroni correction. *Escherichia coli* HT115(DE3) RNA interference (RNAi)‐feeding bacteria were grown overnight in LB medium containing 50 μg/ml ampicillin and 6.25 μg/ml tetracycline in final concentration. L4/young‐stage adults were transferred to plates containing 300–400 mg/ml FUdR, 50 μg/ml ampicillin, 6.25 μg/ml tetracycline, and 0.4 mm IPTG in final concentration. Strains were grown for two generations on RNAi bacteria before assaying for lifespan. After FUdR treatment (about 24–48 hr), animals were transferred to novel RNAi plates. Empty vector‐containing bacteria were used as controls.

### 4.3 Dauer formation assay

L1‐stage larvae were synchronized by isolating eggs from gravid adults. 100–200 embryos were pipetted onto NGM plates seeded with *E. coli OP50* bacteria and kept for 60–72 hr at appropriate temperatures (20–23°C). Animals were well‐fed and maintained at a relatively low population density to avoid dauer formation response triggered by environmental cues. The number of dauer larvae and L4‐stage larvae/adults was determined visually, and scoring was confirmed by SDS (sodium dodecyl sulfate) treatment the dauer larvae are largely resistant for. Animals were considered as dauer larvae if they survived 20 min of incubation in 1% SDS. After SDS treatment, animals were washed with M9 buffer three times, transferred to new NGM plates, and then incubated at 20°C for overnight. The number of surviving animals was determined. Data were analyzed by chi‐squared test with Bonferroni correction.

### 4.4 Reporter constructions and transgenic strains

*daf‐16d/f::gfp* construct contains a 4‐kb‐long upstream regulatory region and the first three exons (0.5 kb) of *daf‐16d/f*, whereas *daf‐16a::gfp* construct contains a 2.4‐kb upstream DNA fragment and the first two exons (1.5 kb) of *daf‐16a*. DNA fragments were amplified by High Fidelity PCR Enzyme Mix (Fermentas) from *C. elegans* genomic DNA template, using the following primers: *daf‐16d/f* forward 5′‐AAA ACT GCA GCC GCC AGC AGA TTT TAT TTG‐3′ and *daf‐16d/f* reverse 5′‐CGC GGA TCC CGC TCT TGT TGA TGG AGG TC‐3′; *daf‐16a* forward 5′‐ACG CGT CGA CAC AAC GTT TTG CCC TTT TTG‐5′ and *daf‐16a* reverse 5′‐CGC GGA TCC TTG TGA CGG ATC GAG TTC TG‐3′. Amplified fragments were digested with BamHI and PstI, and cloned into the vector pPD95.75. For generating _*mut*_ *daf‐16::gfp* reporters (the potential TRA‐1‐binding site was mutated at several critical positions), QuikChange XL Site‐Directed Mutagenesis Kit (Agilent Technologies) was used with the following primers: _*mut*_ *daf‐16d/f* forward 5′‐CTT GGC TCT TCC GTG GTT GCC AGT TGA CAG TT‐3′ and _*mut*_ *daf‐16d/f* reverse 5′‐AAC TGT CAA CRG VCA ACC ACG GAA GAG CCA AG‐3′; _*mut*_ *daf‐16a* forward 5′‐AAT CTG TCA CAA GGC GCA ATG CCG GCA AAA AAA G‐3′ and _*mut*_ *daf‐16a* reverse 5′‐CTT TTT TGC CGG CAT TGC GCC TTG TGA CAG ATT A‐3′. Transgenic strains were generated by microparticle cobombardment. *unc‐119(ed3)* mutant worms were shot with *gfp* reporter constructs along with pRH21 that contains the *unc‐119(+)* rescuing system for the selection of transgenic animals.

### 4.5 Fluorescent microscopy

Transgenic worms were placed on 2% agarose pads and immobilized by adding 0.1 m levamisole in M9 buffer. Pictures in Figure 2c were taken by a Multiphoton Confocal Microscope. Images for quantitative analysis were taken by an Olympus BX‐51 microscope and not overexposed (Figure 2b and Figure S7) or a Zeiss Axio Observer microscope (Figure S6). The software ImageJ was used for quantitative analysis. Expression data were obtained from whole animals. Statistical significance was determined by independent samples *t* tests or Mann–Whitney tests with Bonferroni correction (SPSS 17 software).

### 4.6 Generation of TRA‐1 antibody

A full‐length *tra‐1* cDNA (pDZ118) provided by Dr. David Zarkower (University of Minnesota, US) was used to express a 133 amino acid‐long polypeptide (translated from the 14^th^ exon of *tra‐1*), which was subsequently used as epitope to generate rabbit polyclonal TRA‐1 antibody. The antigen was expressed in QIAexpress system (Qiagen), and 6xHis tag was used for the purification after dialysis. The raised antibody specifically labels a 175‐kDa protein (Figures S9 and S10).

### 4.7 Chromatin immunoprecipitation assays (ChIP)

ChIP was performed as described previously (Ratajewski et al., 2012), with some modifications. Briefly, mixed‐staged animals were collected and grounded to powder under liquid nitrogen, using a sterile mortar and pestle. The resulting worm powder was transferred into cross‐linking buffer (1% formaldehyde in phosphate‐buffered saline; PBS). Fixation time was held for 15 min at room temperature. The reaction was quenched by adding glycine to a final concentration of 0.125 m and sedimented at 17 000 g for 5 min at 4°C. Pellets were washed with PBS four times, then resuspended in Nuclear Lysis Buffer Mix (5 mm PIPES, pH = 8; 85 mm KCl; 0.5% NP40; proteinase inhibitor [Complete cocktail tablets, Roche]), incubated for 15 min at 4°C, and vortexed 20–30 s in every 2 min. After pelleting samples (21 000 g, for 10 min, at 4°C), pellets were resuspended in Sonicating Buffer (1% SDS; 10 mm EDTA; 50 mm Tris‐HCl, pH = 8.1; proteinase inhibitor [Complete cocktail tablets, Roche]). Cross‐linked chromatin was sonicated with maximum power on ice for 9 cycles of 30 s. DNA fragments of approximately 500 bp were determined experimentally by gel electrophoresis. Samples were sedimented at 17 000 g for 5 min at 4°C, and supernatant was transferred into a new tube. Prior to adding antibody, 10% of the volume was taken for the input sample. For each IP reaction, 15–15 μl protein A and G magnetic beads (Life Technologies) were washed in IP buffer:sonicating buffer (9:1) (IP puffer: 0.01% SDS; 1.1% Triton X‐100; 1.2 mm EDTA; 16.7 mm Tris‐HCl (pH = 8.1); 167 mm NaCl, proteinase inhibitor [Complete cocktail tablets, Roche]). For each IP reaction, 1 μg antibody (anti‐TRA‐1; Santa Cruz Biotechnology) and anti‐TRA‐1 antibody generated by our laboratory were added to the beads. Following 2‐hr incubation at 4°C, 500 μl 10× diluted chromatin (in IP buffer:sonicating buffer [9:1]) and DTT (1 m) were added to each reaction. After incubation overnight at 4°C with constant rotation, beads were washed in buffer A (0.1% SDS; 1% Triton X‐100; 2 mm EDTA; 20 mm Tris‐HCl (pH 8.1); 0.15 m NaCl; proteinase inhibitor [Complete cocktail tablets, Roche]), buffer B (0.1% SDS; 1% Triton X‐100; 2 mm EDTA; 20 mm Tris‐HCl (pH = 8.1); 0.5 m NaCl proteinase inhibitor [Complete cocktail tablets, Roche]), buffer C (0.25 m LiCl; 1% NP40; 1% Na‐deoxycholate; 1 mm EDTA; Tris‐HCl (pH 8.1); proteinase inhibitor [Complete cocktail tablets, Roche]) and in TE buffer [10 mm Tris‐HCl (pH 8.0); 10 mm EDTA (pH 8.0)]. After washing, the mixture was incubated for 5 min at 4°C. After the removal of residual TE buffer, 200 μl of elution buffer (0.1 m NaHCO_3_; 1% SDS) was added and the supernatant was transferred into a new tube. For reverse cross‐linking, samples were incubated at 65°C for overnight in 5 m NaCl and 0.5 m EDTA. Following 1‐hr incubation with 1 μl RNaseA (10 mg/ml) at 37°C, 4 μl EDTA, 8 μl TRIS‐HCl (1M, pH 7), and 0.5 μl proteinase K (20 mg/ml, Roche) were added to samples and incubated at 45°C for 2 hr to remove RNA. DNA was purified/recovered using High Pure PCR Template Preparation Kit (11796828001, Roche) and eluted in water. Genomic DNA (input) was prepared by treating aliquots of chromatin with RNaseA, Proteinase K, EDTA, and Tris‐HCl, and heated for decross‐linking, followed by DNA purification.

### 4.8 Measurement of *daf‐16* transcript levels, ChIP, and qRT–PCR

#### 4.8.1 ChIP quantitative PCR

qPCR was used to determine relative amount of specific loci in ChIP vs. input samples. Input and ChIP samples were quantified by real‐time PCR and SYBR Green (LightCycler 480, Roche), using primer pairs specific to the putative TRA‐1‐binding regions. 5 μl of purified ChIP DNA was used in duplicate reactions as a template for amplification using 500 nm of each primer and 10 μl SYBR Green I PCR Master Mix in a 20 μl of total reaction mixture. After denaturation (for 10 min, at 95°C), 45 cycles of amplification (10 s, 95°C; 10 s, 60°C; and 20 s, 72°C) were performed. The TRA‐1‐binding site in the *xol‐1* gene was chosen for positive control. A locus negative for TRA‐1 binding (*daf‐11*) was used as an internal control to normalize quantification in qPCRs. Forward and reverse primer sequences (designed by the BiSearch software; Arányi, Váradi, Simon & Tusnády, 2006) used for qPCR were as follows. *xol‐1* (positive control): 5′‐GAA TAC CCC TGT AAG ACC ACA CA‐3′ and 5′‐AGG ACG CAG ACA CGT TAG AAT AG‐3′; *daf‐11* (negative control): 5′‐CCT TAA TCC CTG CAC ACG TT‐3′ and 5′‐CCG AGC AAA AAC AAT GAT GA‐3′; *daf‐16d/f*: 5′‐CAA GCC TCA AAC ACC AGT GA‐3′ and 5′‐CTG TCAA CTG GCA AGA CCA C‐3′; *daf‐16a*: 5′‐TGC AAC AAA TTC CTC TCA ACA G‐3′ and 5′‐GCT TCT TAC GAC AAC GCT TCT T‐3′. Normalized data were analyzed by independent samples *t* tests with Bonferroni correction (SPSS17 software).

#### 4.8.2 RNA extraction and quantitative real‐time PCR

Total RNA was extracted from approximately 30‐30 synchronized L4/young (nongravid) adult animals using ChargeSwitch Total RNA Cell Kit (CS14010, Invitrogen). For quantitative real‐time PCR analysis, total RNA was used for first‐strand cDNA synthesis by RevertAid First Strand cDNA Synthesis Kit (K1622, Thermo Scientific). Real‐time PCR was performed on LightCycler Carousel Detection System (Roche) and LightCycler 480 System (Roche), using the following conditions: denaturation: 95°C for 10 min, followed by 45 cycles of amplification (10 s, 95°C; 10 s, 58°C; 20 s, 72°C). Specific PCR products were detected by the fluorescence of double‐stranded DNA‐binding SYBRGreen dye. Melting curve analysis was performed to confirm correct PCR product size and absence of nonspecific bands. Relative mRNA levels were determined by normalizing the PCR threshold cycle number of *daf‐16* with that of *ama‐1* or *pmp‐3* reference genes. Forward and reverse primers were as follows: *pmp‐3*: 5′‐GTT CCC GTG TTC ATC ACT CAT‐3′ and 5′‐ACA CCG TCG AGA AGC TGT AGA‐3′; *ama‐1*: 5′‐GAA GGT CGC AGG TGG ATG‐3′ and 5′‐GCA TGA TTT TTC GCT CCT G‐3′; *daf‐16d/f*: 5′‐CTC GTT CTC TCC GTA TTT CCA C‐3′ and 5′‐TGT CCA CAT TGC TCA TTG CT‐3′; *daf‐16a*: 5′‐GAA CGA TCT AGT CCC GAG GAG‐3′ and 5′‐TTC TGA ATT CGC ATG AAA CG‐3′. Data were analyzed with Pair Wise Fixed Reallocation Randomization Test, REST program.

## DECLARATION

The authors declare no competing financial interest.

## AUTHOR CONTRIBUTIONS

B.Ha. and T.V. invented the project; B.Ho. designed and performed lifespan measurements, generated transgenic strains, monitored dauer development, performed quantitative real‐time PCR and Western blot analysis, and analyzed data; M.K. performed lifespan assays, monitored dauer development, analyzed ChIP data, did quantitative real‐time PCR, and generated transgenic strains; B.Ha. identified TRA‐1‐binding sites in the *daf‐16* locus, generated a TRA‐1‐specific antibody and transgenic strains, performed lifespan measurements, analyzed data, and wrote the manuscript; M.L. generated the TRA‐1‐specific antibody; K.T‐V. designed experiments, analyzed data, and wrote the manuscript; J.B. identified conserved TRA‐1‐binding sites in closely related nematode taxa, performed lifespan measurements, and analyzed data; K.B. analyzed *gfp* expression and performed lifespan measurements; A.M‐C. designed experiments, analyzed data, and wrote the manuscript; C.O. performed in silico analyses to identify GLI binding sites in *FOXO* genomic regions; C.B. performed ChIP experiments and transcript quantification; A.P. performed fluorescent microscopy analyses; T.A. designed experiments, analyzed data, and wrote the manuscript; N.T. designed experiments, analyzed data, and wrote the manuscript; and T.V. designed experiments, analyzed data, and wrote the manuscript. All authors agree with the presented findings.
