# Supplementary material for: Sex‐specific regulation of aging in Caenorhabditis elegans
Source: Aging Cell. 2018 Mar 1;17(3):e12724. doi: 10.1111/acel.12724 (PMC5946081; doi:10.1111/acel.12724)
Supplement: Supplementary file 1 [file ACEL-17-e12724-s001.docx]

**Supporting Information**

**Sex-specific Regulation of Aging in *Caenorhabditis elegans***

**Bernadette Hotzi, Mónika Kosztelnik, Balázs Hargitai, Krisztina Takács-Vellai, János Barna, Kincső Bördén, András Málnási-Csizmadia, Csaba Ortutay, Mónika Lippai, Caroline Bacquet, Angela Pasparaki, Tamás Arányi, Nektarios Tavernarakis, and Tibor Vellai**

**Supporting Information**

**Supporting Experimental Procedures**

**Supplemental References (1 - 10)**

**Tables S1 to S8**

**Figures S1 to S12**

**Supplemental Experimental Procedures**

**Western blot analysis**

Protein samples prepared from 200 young adult worms were resolved on SDS-PAGE, and transferred to a nitrocellulose membrane. Membranes were probed with anti-TRA-1 (rabbit, 1:1000), anti-alpha-Tub84B (mouse, 1:2500, Sigma, T6199), anti-rabbit IgG alkaline phosphatase (1:1000, Sigma, A3687) and anti-mouse IgG alkaline phosphatase (1:1000, Sigma, A8438) antibodies, and developed by NBT-BCIP solution (Sigma, 72091).

**Identification of conserved GlI binding sites in the genomic region of Human *FOXOs***

Conserved GLI binding sites in the genomic region of the human *FOXO* genes.

*GLI binding motifs.* The GLI (Glioma-associated) family zinc finger transcription factors have been studied for a long time and their DNA binding specificity was described in different conditions. Hallikas *et al.* (2006) published a consensus binding site with a sequence of GACCACCCA as the most representative GLI motif (Hallikas *et al*., 2006). Because of the slightly degenerate nature of the binding site, a more specific representation can be achieved using the following position-specific weight matrix (PSWM):

A 9 758 30 0 813 0 166 25 964

C 4 136 971 1000 74 1000 823 941 9

G 945 106 0 0 24 0 0 2 6

T 42 0 0 0 89 0 11 32 22

This motif was used as a starting point in the following analysis. Since the motif is rather short, it is expected to deliver many false positive hits. To circumvent this, this motif was used to query MotifDB for further, more exact binding site information. Based on the results of this search, two further binding patterns were identified: Gli2 and GLI2-2 (Jolma *et al*., 2013; Gentleman *et al*., 2014) (**Fig. S11**). These three sequence patterns were all used in the binding site prediction calculations.

*GLI target genes*. In the human genome, there are six members of the Forkhead box O gene family (FOXO), which are the subjects of the present binding site analysis: *FOXO1*, *FOXO1b*, *FOXO3*, *FOXO3b*, *FOXO4*, and *FOXO6*. Out of them, FOXO1b and FOXO3b are annotated as processed pseudogenes in the Entrez Gene database (Maglott *et al.,* 2005), therefore, they were excluded from further analyzes here.

Next we considered the conservation of the identified sites. Since FOXO4 seems to be specific to the Primates group, no suitable more distant orthologues were possible to be identified in the Ensembl database (Flicek *et al*., 2014). For this reason, *FOXO4* was also excluded from the present binding site prediction analysis. After these considerations, the genomic regions of *FOXO1*, *FOXO3*, and FOXO6 were submitted to motif search. The genomic regions of the selected three genes were obtained from the Ensembl database. The intergenic genomic region upstream and downstream of these *FOXO* genes was considered as follows: for *FOXO1* block1 (alignment): Chromosome 13 40547762-40614067 (Genomic region in GRCh38 genome build); for *FOXO1* block6: Chromosome 13 40538711-40547761; for *FOXO3* promoter region: Chromosome 6 108527366-108562796; for *FOXO3* gene region: Chromosome 6 108562797-108697835. The eutherian orthologue alignments of these regions were obtained from Ensembl. For the motif search Motif Occurrence Detection Suite (Korhonen *et al*., 2009) was applied on the human sequences from each alignment using custom written BioPerl6 scripts. The number of hits from the genomic regions of the genes: GLI consensus (motif): 26 (FOXO1), 100 (FOXO3), 18 (FOXO3 promoter), 14 (FOXO6); GLI2 Jolma 2013 (motif): 23 (FOXO1), 112 (FOXO3), 20 (FOXO3 promoter), 8 (FOXO6); GLI2-2 Jolma2013 (motif): 12 (FOXO1), 48 (FOXO3), 6 (FOXO3 promoter), 6 (FOXO6). As can be seen, the searches revealed a much higher number of hits in the region of *FOXO3* genes compared to the others, therefore only hits in the promoter region of this gene were investigated further (the numbers are shown in the separate column). The conservation of hits in the eutherian orthologs with scores above 9 was investigated manually using software Jalview (Waterhouse *et al.,* 2009). In the case of *FOXO6*, the entire region is less conserved. Most of the high score hits are not conserved in any of the genes. The only exceptions are two GLI2 hits in the known promoter region of *FOXO3* gene. The hits with P<0.0001 in the promoter regions of *FOXO3* gene were imported to the Ensembl genome browser, and the conservation of the region of the later site was visualized using Seaview alignment viewer (Gouy *et al*., 2010) (**Fig. S12**).

**Supporting References**

Flicek P, Amode MR, Barrell D, Beal K, Billis K, Brent S, Carvalho-Silva D, Clapham P, Coates G, Fitzgerald S, *et al.* (2014) Ensembl 2014. *Nucleic Acids Res*. **42**, D749–755.

Gentleman RC, Carey VJ, Bates DM, Bolstad B, Dettling M, Dudoit S, Ellis B, Gautier L, Ge Y, Gentry J, et al. (2004) Bioconductor: open software development for computational biology and bioinformatics. *Genome Biol*. **5**, R80.

Gouy M, Guindon S, Gascuel O (2010) SeaView version 4: A multiplatform graphical user interface for sequence alignment and phylogenetic tree building. *Mol. Biol. Evol*. **27**, 221–224.

Hallikas O, Palin K, Sinjushina N, Rautiainen R, Partanen J, Ukkonen E, Taipale J (2006) Genome-wide prediction of mammalian enhancers based on analysis of transcription-factor binding affinity. *Cell* **124**, 47–59.

Jolma A, Yan J, Whitington T, Toivonen J, Nitta KR, Rastas P, Morgunova E, Enge M, Taipale M, Wei G, *et al.* (2013) DNA-binding specificities of human transcription factors. *Cell* **152**, 327–339.

Korhonen J, Martinmäki P, Pizzi C, Rastas P, Ukkonen E (2009) MOODS: fast search for position weight matrix matches in DNA sequences. *Bioinforma. Oxf. Engl*. **25**, 3181–3182.

Kwon ES, Narasimhan SD, Yen K, Tissenbaum HA (2010) A new DAF-16 isoform regulates

longevity. *Nature* **466**, 498–502.

Maglott D, Ostell J, Pruitt KD, Tatusova T (2005) Entrez Gene: gene-centered information at NCBI. *Nucleic Acids Res*. **33**, D54–D58.

Segal SP, Graves LE, Verheyden J, Goodwin EB (2001) RNA-regulated TRA-1 nuclear export controls sexual fate. *Dev. Cell* **1**, 539–551.

Waterhouse AM, Procter JB, Martin DMA, Clamp M, Barton GJ (2009) Jalview Version 2--a multiple sequence alignment editor and analysis workbench. *Bioinforma. Oxf. Engl*. **25**, 1189–1191.

**Supporting Tables**

**Table S1. Statistics for life span data.** Animals were maintained at 25°C, otherwise indicated.

| **Genotype** | **Number of worms** | **Number of plates** | **Mean lifespan (days)** | **±S.E.M. (days)** | **Log Rank P value (with Bonferroni correction)** | **Independent *t*-test P value (with Bonferroni correction)** |
| --- | --- | --- | --- | --- | --- | --- |
| for **Figures 1A, A’, S1, S2** | | | | | | |
| *wild-type herm.* | 526 | 9 | 11.70 | 0.06 |  |  |
| *wild-type male* | 148 | 9 | 10.16 | 0.15 | vs. *wild-type herm*.  P < 0.0001 | vs. *wild-type herm*.  P < 0.0001 |
| *wild-type herm.* (20°C) | 182 | 4 | 15.25 | 0.23 |  |  |
| *wild-type male*  (20°C) | 31 | 4 | 12.65 | 0.41 | vs. *wild-type herm*. (20°C)  P < 0.0001 | vs. *wild-type herm*.  (20°C)  P < 0.0001 |
| *wild-type herm.* (without FUdR) | 675 | 15 | 9.15 | 0.15 |  |  |
| *wild-type male* (without FudR) | 75 | 15 | 7.8 | 0.2 | vs. *wild-type herm*. (without FUdR)  P < 0.0001 | vs. *wild-type herm*.  (without FUdR)  P < 0.0001 |
| for **Figure 1C, C’** | | | | | | |
| *daf-2(e1370) herm.* | 432 | 7 | 24.56 | 0.15 |  |  |
| *daf-2(e1370) male* | 170 | 7 | 21.62 | 0.21 | vs. *daf-2(e1370*) *herm*. P < 0.0001 | vs. *daf-2(e1370*) *herm*.  P < 0.0001 |
| *daf-2(e1370);*  *daf-16(mu86) herm.* | 500 | 8 | 8.29 | 0.07 | vs. *daf-2(e1370*) *herm*. P < 0.0001 | vs. *daf-2(e1370*) *herm*.  P < 0.0001 |
| *daf-2(e1370);*  *daf-16(mu86) male* | 138 | 8 | 8.06 | 0.11 | vs. *daf-2(e1370*);  *daf-16(mu86) herm*.  P = 0.0996 | vs. *daf-2(e1370*);  *daf-16(mu86) herm*.  P = 0.4329 |
| for **Figure 1E, E’** | | | | | | |
| *wild type* | 118 | 2 | 11.31 | 0.10 |  |  |
| *tra-1(e1099)* | 98 | 2 | 8.76 | 0.22 | vs. *wild-type*  P < 0.0001 | vs. *wild-type*  P < 0.0001 |
| *tra-1(e1488)* | 71 | 2 | 8.03 | 0.20 | vs. *wild-type*  P < 0.0001 | vs. *wild-type*  P < 0.0001 |
| *fem-3(e2006)* | 148 | 2 | 12.39 | 0.20 | vs. *wild-type*  P < 0.0001 | vs. *wild-type*  P < 0.0001 |
| *tra-3(e1767)* | 102 | 2 | 7.17 | 0.17 |  |  |
| *tra-1(e1575gf)/+;*  *tra-3(e1767)* | 96 | 2 | 10.93 | 0.28 | vs. *tra-3(e1767)*  P < 0.0001 | vs. *tra-3(e1767)*  P < 0.0001 |
| for **Figure 1F, F’** | | | | | | |
| *wild-type* | 145 | 2 | 12.73 | 0.15 |  |  |
| *daf-16(mu86)* | 110 | 2 | 10.63 | 0.12 | vs. *wild-type*  P < 0.0001 | vs. *wild-type*  P < 0.0001 |
| *fem-3(e2006)* | 129 | 2 | 15.05 | 0.31 | vs. *wild-type*  P < 0.0001 | vs. *wild-type*  P < 0.0001 |
| *fem-3(e2006);*  *daf-16(mu86)* | 105 | 2 | 10.54 | 0.16 | vs. *daf-16(mu86*)  P = 4.6577 | vs. *daf-16(mu86*)  P = 4.0016 |
|  |  |  |  |  | vs. *fem-3(e2006)*  P < 0.0001 | vs. *fem-3(e2006)*  P < 0.0001 |
| for **Figure 1G, G’** | | | | | | |
| *wild-type (ev)* | 104 | 2 | 12.35 | 0.19 |  |  |
| *daf-16(RNAi)* | 44 | 2 | 9.41 | 0.22 | vs. *wild-type (ev)*  P < 0.0001 | vs. *wild-type (ev)*  P < 0.0001 |
| *fem-3(e2006) (ev)* | 151 | 2 | 13.58 | 0.24 | vs. *wild-type (ev)*  P < 0.0001 | vs. *wild-type (ev)*  P = 0.0025 |
| *fem-3(e2006);*  *daf-16(RNAi)* | 109 | 2 | 8.83 | 0.12 | vs. *daf-16(RNAi*)  P = 0.0583 | vs. *daf-16(RNAi*)  P = 0.0903 |
|  |  |  |  |  | vs. *fem-3(e2006) (ev)*  P < 0.0001 | vs. *fem-3(e2006) (ev)*  P < 0.0001 |
| for **Figure 1H, H’** | | | | | | |
| *daf-16(mu86)* | 131 | 2 | 9.50 | 0.07 |  |  |
| *tra-3(e1767)* | 161 | 3 | 7.70 | 0.14 |  |  |
| *tra-1(e1575)/+;*  *tra-3(e1767)* | 137 | 3 | 11.20 | 0.20 | vs. *daf-16(mu86*)  P < 0.0001 | vs. *daf-16(mu86*)  P < 0.0001 |
|  |  |  |  |  | vs. *tra-3(e1767)*  P < 0.0001 | vs. *tra-3(e1767)*  P < 0.0001 |
| *tra-1(e1575)/+;*  *tra-3(e1767);*  *daf-16(mu86)* | 137 | 3 | 7.00 | 0.07 | vs. *tra-3(e1767)*  P < 0.0001 | vs. *tra-3(e1767)*  P < 0.0001 |
|  |  |  |  |  | vs. *tra-1(e1575)/+;*  *tra-3(e1767)*  P < 0.0001 | vs. *tra-1(e1575)/+;*  *tra-3(e1767)*  P < 0.0001 |
| for **Figure 1I, I’** | | | | | | |
| *daf-16(RNAi)* | 42 | 2 | 9.40 | 0.22 |  |  |
| *tra-3(e1767) (ev)* | 83 | 2 | 7.69 | 0.20 |  |  |
| *tra-1(e1575)/+;*  *tra-3(e1767) (ev)* | 88 | 2 | 10.51 | 0.24 | vs. *daf-16(RNAi*)  P = 0.0002 | vs. *daf-16(RNAi*)  P = 0.0282 |
|  |  |  |  |  | vs. *tra-3(e1767)* (ev)  P < 0.0001 | vs. *tra-3(e1767)* (ev)  P < 0.0001 |
| *tra-1(e1575)/+;*  *tra-3(e1767);*  *daf-16(RNAi)* | 35 | 2 | 8.80 | 0.27 | vs. *tra-3(e1767)* (ev)  P = 0.0654 | vs. *tra-3(e1767)* (ev)  P = 0.0112 |
|  |  |  |  |  | vs. *tra-1(e1575)/+;*  *tra-3(e1767) (ev)*  P < 0.0001 | vs. *tra-1(e1575)/+;*  *tra-3(e1767) (ev)*  P < 0.0001 |
| for **Figure S3** | | | | | | |
| *daf-2(e1370) herm.* | 366 | 5 | 25.00 | 0.19 |  |  |
| *daf-2(e1370) male* | 74 | 5 | 22.41 | 0.52 | vs. *daf-2(e1370*) *herm*. P < 0.0001 | vs. *wild-type herm*.  P < 0.0001 |
| *daf-2(e1370);*  *daf-16(mgDf50) herm.* | 290 | 4 | 9.37 | 0.09 | vs. *daf-2(e1370*) *herm*. P < 0.0001 | vs. *wild-type herm*.  P < 0.0001 |
| *daf-2(e1370);*  *daf-16(mgDf50) male* | 71 | 4 | 9.37 | 0.17 | *vs*. *daf-2(e1370);*  *daf-16(mgDf50) herm*.  P = 0.827 | vs. *daf-2(e1370);*  *daf-16(mgDf50)* *herm*.  P = 5.9332 |
| for **Figure 3** | | | | | | |
| *wild-type male*  *(60 herms. : 10 males)* | 75 | 7 | 10.13 | 0.21 |  |  |
| *daf-16(mgDf50) male*  *(60 herms. : 10 males)* | 102 | 8 | 7.61 | 0.1 | vs. *wild-type* male  P < 0.0001 | vs. *wild-type* male  P < 0.0001 |
| *daf-16(mgDf50); Ip1s14 (daf-16d/f::gfp) male*  *(60 herms. : 10 males)* | 65 | 6 | 10.65 | 0.24 | vs. *wild-type* males  P = 0.408  vs. *daf-16(mgDf50)* male  P < 0.0001 | vs. *wild-type* male  P = 0.325  vs. *daf-16(mgDf50)* male  P < 0.0001 |
| *wild-type herm.*  *(60 herms. : 10 males)* | 410 | 7 | 12.2 | 0.11 |  |  |
| *daf-16(mgDf50) herm.*  *(60 herms. : 10 males)* | 568 | 8 | 9.16 | 0.05 | vs. *wild-type* herm.  P < 0.0001 | vs. *wild-type* herm.  P < 0.0001 |
| *daf-16(mgDf50); Ip1s14 (daf-16d/f::gfp) herm.*  *(60 herms. : 10 males)* | 302 | 6 | 13.68 | 0.1 | vs. *wild-type* herm.  P < 0.0001  vs. *daf-16(mgDf50)* herm.  P < 0.0001 | vs. *wild-type* herm.  P < 0.0001  vs. *daf-16(mgDf50)* herm.  P < 0.0001 |
| for **Figure S8** | | | | | | |
| *wild-type male*  *(30 males/plate)* | 57 | 2 | 8.81 | 0.22 |  |  |
| *daf-16(mgDf50) male*  *(30 males/plate)* | 65 | 2 | 6.32 | 0.08 | vs. *wild-type* male  P < 0.0001 | vs. *wild-type* male  P < 0.0001 |
| *daf-16(mgDf50); Ip1s14 (daf-16d/f::gfp) male*  *(30 males/plate)* | 54 | 2 | 9.67 | 0.27 | vs. *wild-type* males  P = 0.045  vs. *daf-16(mgDf50)* male  P < 0.0001 | vs. *wild-type* male  P = 0.015  vs. *daf-16(mgDf50)* male  P < 0.0001 |

**Table S2. Statistics for the relative expression intensity of daf-16::gfp reporters.**

| **Transgene** | **Background** | **Number of worms** | **Mean of relative expression level** | **±S.E.M.** | **Independent samples *t*-test P value vs. control** |
| --- | --- | --- | --- | --- | --- |
| for **Figure 2D** | | | | | |
| *daf-16d/f/h::gfp* | XX herm. | 250 | 1.000 | 0.014 |  |
|  | XO male | 43 | 0.664 | 0.020 | vs. herm.  P < 0.0001 |
|  | *tra-1(e1099)* | 97 | 0.758 | 0.027 | vs. herm.  P < 0.0001  vs. male  P = 0.056 |
|  | *tra-1(e1488)* | 206 | 0.639 | 0.014 | vs. herm.  P < 0.0001  vs. male  P = 3.047 |
|  | *fem-3(e2006)* | 46 | 1.182 | 0.042 | vs. herm.  P < 0.0001 |
| for **Figure 2E** | | | | | |
| *_mut_daf-16d/f/h::gfp* | XX herm. | 233 | 1.000 | 0.011 |  |
|  | XO male | 9 | 1.029 | 0.052 | vs. herm.  P = 3.841 |
|  | *tra-1(e1099)* | 71 | 0.998 | 0.019 | vs. herm.  P = 5.594  vs. male  P = 0.186 |
|  | *tra-1(e1488)* | 75 | 0.970 | 0.018 | vs. herm.  P = 0.838  vs. male  P = 2.190 |
|  | *fem-3(e2006)* | 31 | 1.030 | 0.024 | vs. herm.  P = 1.928 |
| for **Figure 2F** | | | | | |
| *daf-16a::gfp* | *control* | 28 | 1.000 | 0.071 | control |
|  | *tra-1(e1488)* | 17 | 0.705 | 0.081 | 0.0111 |
|  | *fem-3(e2006)* | 13 | 1.485 | 0.167 | 0.0031 |
| for **Figure 2G** | | | | | |
| *_mut_daf-16a::gfp* | *control* | 39 | 1.001 | 0.084 | control |
|  | *tra-1(e1488)* | 26 | 1.127 | 0.192 | 0.4995 |
|  | *fem-3(e2006)* | 16 | 0.917 | 0.098 | 0.0500 |
| for **Figure 3C, C’** | | | | | |
| *daf-16d/f::gfp (lpIs14)* | XX herm. | 16 | 1.000 | 0.026 |  |
|  | XO male | 14 | 0.694 | 0.035 | 0.0001 |

**Table S3. Statistics for dauer formation assays.**

| **Temperature** | **Genotype** | **Number of worms** | **Number of  plates** | **dauer %** | **Chi-squared test P value vs. control (with Bonferroni correction)** |
| --- | --- | --- | --- | --- | --- |
| for **Figure 2H** | | | | | |
| 20°C | *daf-2(e1368)* | 3812 | 27 | 0.2 | control |
|  | *fem-3(e2006);daf-2(e1368)* | 3006 | 24 | 2.8 | 0.0000 |
|  | *daf-2(e1370)* | 2475 | 25 | 3.4 | control |
|  | *tra-1(e1488);daf-2(e1370)* | 1575 | 8 | 0.1 | 0.0000 |
|  | *fem-3(e2006); daf-2(e1370)* | 2001 | 37 | 17.4 | 0.0000 |
| for **Figure 2I** | | | | | |
| 23°C | *daf-2(e1370)* | 194 | 2 | 27.3 | control |
|  | *tra-1(e1488); daf-2(e1370)* | 452 | 2 | 16.7 | 0.0060 |
|  | *fem-3(e2006); daf-2(e1370)* | 142 | 2 | 47.2 | 0.0005 |

**Table S4. Statistics for ChIP data. “**Ab”, antibody. *daf-11* serves as a negative control*, xol-1* serves as a positive control (a known target gene).

| **Antibody** | **%input** | **SD** | **% input** | **±S.D.** | **Independent samples *t*-test P value *daf-11* vs. target gene** |
| --- | --- | --- | --- | --- | --- |
| for **Figure 4A** |  |  |  |  |  |
|  | *daf-11* |  | *xol-1* |  |  |
| *mouse IgG* | 0.510 | 0.014 | 0.485 | 0.227 | 0.889 |
| *TRA-1 Ab (Vellai lab.)* | 0.754 | 0.068 | 1.730 | 0.028 | 0.003 |
| *TRA-1 Ab (com.)* | 0.148 | 0.031 | 0.931 | 0.013 | 0.001 |
| for **Figure 4B** |  |  |  |  |  |
|  | *daf-11* |  | *daf-16d/f/h* |  |  |
| *mouse IgG* | 0.354 | 0.041 | 0.348 | 0.037 | 0.902 |
| *TRA-1 Ab (Vellai lab.)* | 0.501 | 0.145 | 0.875 | 0.055 | 0.026 |
| *TRA-1 Ab (com.)* | 0.380 | 0.064 | 0.888 | 0.033 | 0.002 |
| for **Figure 4C** |  |  |  |  |  |
|  | *daf-11* |  | *daf-16a* |  |  |
| *mouse IgG* | 1.267 | 0.176 | 1.164 | 0.037 | 0.505 |
| *TRA-1 Ab (Vellai lab.)* | 1.014 | 0.195 | 2.757 | 0.515 | 0.004 |
| *TRA-1 Ab (com.)* | 1.031 | 0.295 | 2.057 | 0.425 | 0.009 |

**Table S5. Statistics for qRT-PCR data.** “herm.” indicates hermaphrodites.

| **Gene** | **Stage** | **Background** | **Mean of Relative mRNA level** | **±S.D.** | **Pair Wise Fixed Reallocation Randomization test P value vs. control** |
| --- | --- | --- | --- | --- | --- |
| *daf-16d/f/h* | adult | **Figure 4D** | | | |
|  |  | herm. | 1.000 | control | control |
|  |  | male | 0.509 | 0.181 | 0.049 |
|  |  | **Figure 4E** | | | |
|  |  | *tra-1(e1575)/+;tra-3(e1767)* | 1.000 | control | control |
|  |  | *tra-3(e1767)* | 0.663 | 0.133 | 0.032 |
| *daf-16a* | L1 | **Figure 4F** | | | |
|  |  | *daf-2(e1370)* | 1.000 | control | control |
|  |  | *tra-1(e1488); daf-2(e1370)* | 0.760 | 0.281 | 0.007 |
|  |  | *fem-3(e2006); daf-2(e1370)* | 7.781 | 1.512 | 0.000 |
|  | dauer | **Figure 4G** | | | |
|  |  | *daf-2(e1370)* | 1.000 | control | control |
|  |  | *tra-1(e1488); daf-2(e1370)* | 0.470 | 0.318 | 0.000 |
|  |  | *fem-3(e2006); daf-2(e1370)* | 2.732 | 1.613 | 0.004 |

**Table S6. Statistics for qRT-PCR data.** “herm.” indicates hermaphrodites.

|  | **Stage** | **Background** | **Mean of Relative mRNA level** | **±S.D.** | **Pair Wise Fixed Reallocation Randomization Test P value vs. control** |
| --- | --- | --- | --- | --- | --- |
| *daf-16d/f/h* | 1-day- old adult | **Figure S5** | | | |
|  |  | XX herm. | 1.000 | 0.161 | control |
|  |  | XO male | 0.531 | 0.149 | 0.020 |
|  |  | *tra-1(1099)* XX male | 0.535 | 0.133 | 0.018 |
|  |  | *tra-1(e1488)* XX male | 0.768 | 0.212 | 0.205 |
| *daf-16d/f/h* | L4 | **Figure S5** | | | |
|  |  | XX herm. | 1.000 | 0.133 | control |
|  |  | XO male | 0.747 | 0.085 | 0.0499 |
|  |  | *tra-1(1099)* XX male | 0.382 | 0.076 | 0.002 |
|  |  | *tra-1(e1488)* XX male | 0.815 | 0.340 | 0.432 |

**Table S7. Statistics for *daf-16d/f* expression at L4 larval, as well as young and old adult stages.** “herm.” indicates hermaphrodite.

| **Transgene (developmental /adult stage)** | **Background** | **Number of worms** | **Trials** | **Mean of relative expression level** | **±S.E.M.** | **Mann-Whitney Test P value (with Bonferroni correction)** |
| --- | --- | --- | --- | --- | --- | --- |
| *daf-16d/f/h::gfp*  *old adult (6-day-old)* | *him-5(e1490) herm.* | 66 | 2 | 1.000 | 0.036 |  |
|  | *him-5(e1490) male* | 62 | 2 | 0.767 | 0.026 | vs. *him-5(e1490)* herm. P < 0.0001 |
|  | *tra-1(e1099)* | 69 | 2 | 0.846 | 0.037 | vs. *him-5(e1490)* herm. P = 0.007  vs. *him-5(e1490)* male P = 6.056 |
|  | *tra-1(e1488)* | 66 | 2 | 1,179 | 0.053 | vs. *him-5(e1490)* herm. P = 0.205  vs. *him-5(e1490)* male P < 0.0001 |

**Table S8. Statistics for *daf-16a* expression at L4 larval, as well as young and old adult stages.** “herm.” denotes hermaphrodite.

| **Transgene (stage)** | **Genotype/sex** | **Number of worms** | **Mean of relative expression level** | **±SEM** | **Mann-Whitney-test P value (with Bonferroni correction)** |
| --- | --- | --- | --- | --- | --- |
| *daf-16a::gfp*  (*L4*) | herm. | 34 | 1.000 | 0.046 |  |
|  | male | 44 | 2.154 | 0.094 | vs. herm. P<0.0001 |
|  | *tra-1(e1099)* | 24 | 2.244 | 0.250 | vs. herm. P<0.0001 vs. male P=4.549 |
|  | *tra-1(e1488)* | 26 | 1.566 | 0.104 | vs. herm. P<0.0001 vs. male P=0.002 |
|  | | | | | |
| *daf-16a::gfp*  (*1-day-old adult*) | herm. | 41 | 1.000 | 0.048 |  |
|  | male | 39 | 2.491 | 0.070 | vs. herm. P<0.0001 |
|  | *tra-1(e1099)* | 30 | 1.644 | 0.216 | vs. herm. P=0.148  vs. male p<0.0001 |
|  | *tra-1(e1488)* | 11 | 1.961 | 0.274 | vs. herm. P<0.0001 vs. male P=0.041 |
|  | | | | | |
| *daf-16a::gfp*  (*6-day-old adult*) | herm. | 31 | 1.000 | 0.067 |  |
|  | male | 25 | 1.994 | 0.200 | vs. herm. P<0.0001 |
|  | *tra-1(e1099)* | 54 | 1.379 | 0.077 | vs. herm. P=0.005  vs. male P=0.010 |
|  | *tra-1(e1488)* | 11 | 1.338 | 0.069 | vs. herm. P=0.018  vs. male P=0.070 |

**Supporting Figures and Figure Legends (Figs. S1-S8)**

**Figure S1.**

**
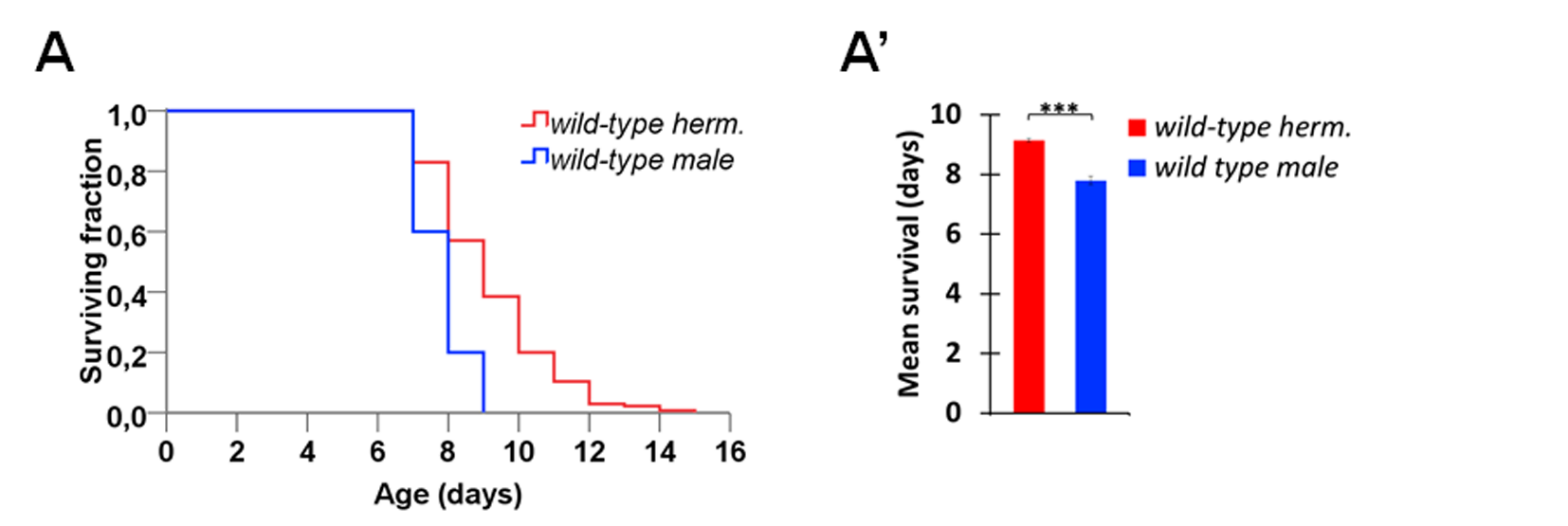
**

**Figure S1. Wild-type hermaphrodites live longer than males, when maintained on media without FUdR.** (**A**) Kaplan-Meyer life span curves, (**A’**) the corresponding mean survival data. “herm.” denotes hermaphrodites. *** indicates P<0.001, Log Rank and Independent samples *t*-test with Bonferroni correction. For statistics and data, see **Table S1**. FUdR: 5-fluoro-2-deoxyuridine. Animals were maintained at 25°C.

**Figure S2.**


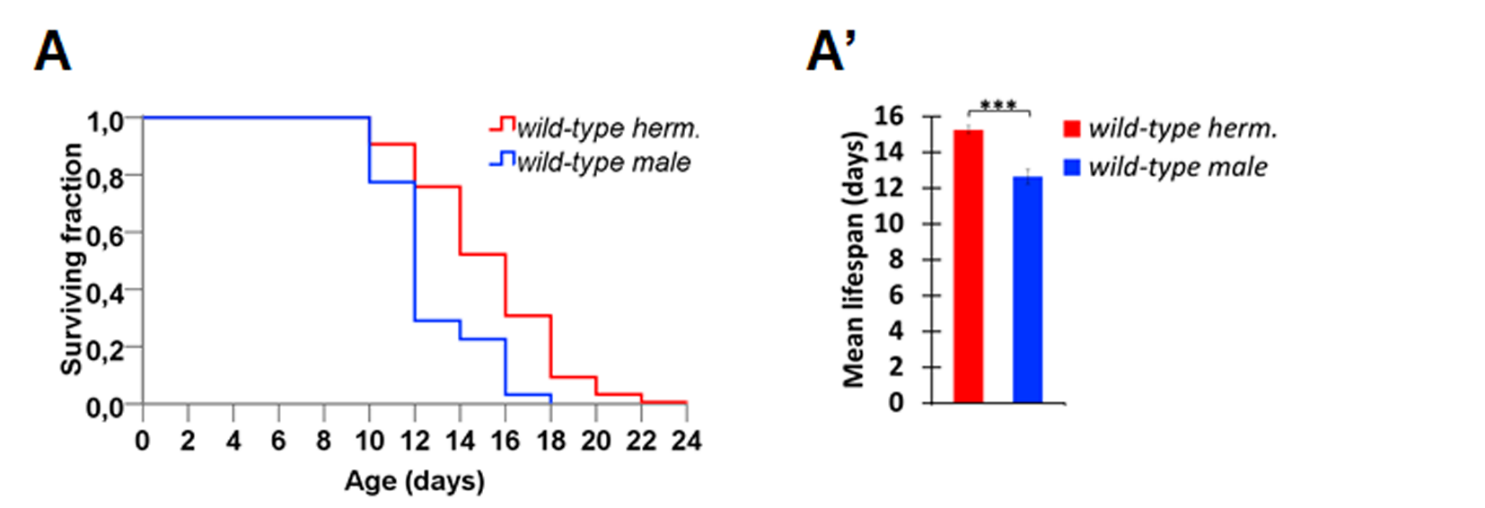


**Figure S2. In populations containing both sexes, hermaphrodites live longer than males at 20°C**. (**A**) The life span curve of wild-type hermaphrodites (red) and males (blue) at 20°C. (**A’**) Mean life span data of wild-type hermaphrodites and males. “herm.” indicates hermaphrodite. *** indicates P<0.001, Log Rank and Independent samples *t*-test with Bonferroni correction. For statistics and data, see **Table S1**.

**Figure S3.**

**
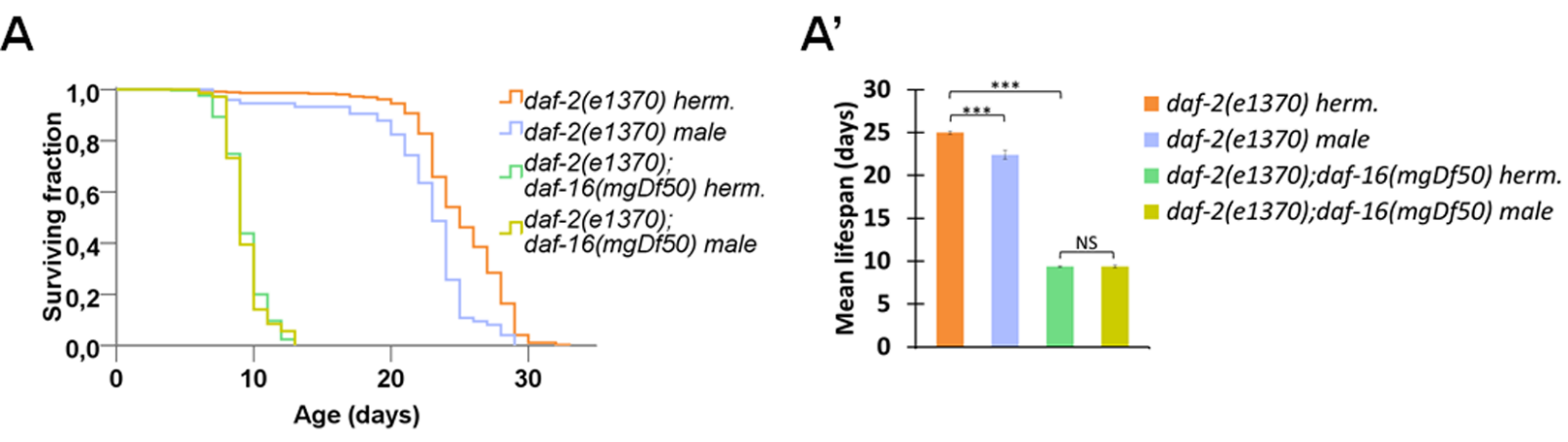
**

**Figure S3. The longer life span of *daf-2(e1370)* mutant hermaphrodites over males is suppressed by *daf-16(mgDf50)* mutation.** (**A**) Kaplan-Meyer life span curves, (**A’**) the corresponding mean life span data. “herm.” denotes hermaphrodites; NS, statistically not significant. *** indicates P<0.001, Log Rank and Independent samples *t*-test. For statistics and data, see also **Table S1**. Animals were maintained at 20°C until they developed into the L4 larval stage, then transferred at 25°C.

**Figure S4.**

**
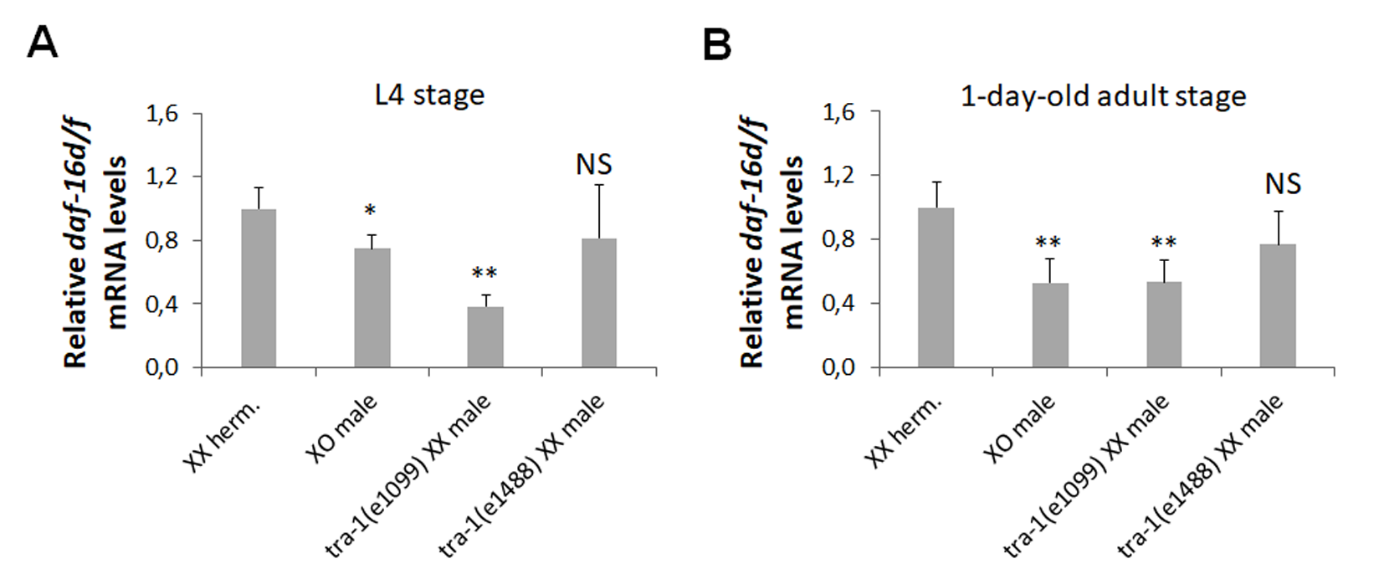
**

**Figure S4. Relative *daf-16d/f* transcript levels at the L4 larval and 1-day-old adult stages.** (**A**) qRT-PCR analysis showing relative *daf-16d/f* transcript levels at L4 larval stages. *cdc-42* was used as an internal control. (**B**) qRT-PCR analysis displaying relative *daf-16d/f* transcript levels at a young (1-day-old) adulthood. *pmp-3* was used as an internal control. In panels (**A** and **B**), bars represent ±S.D., *: P<0.05; **: P<0.01; NS: not significant; Pair Wise Fixed Reallocation Randomization test. For statistics and data, see **Table S6**.

**Figure S5.**

**
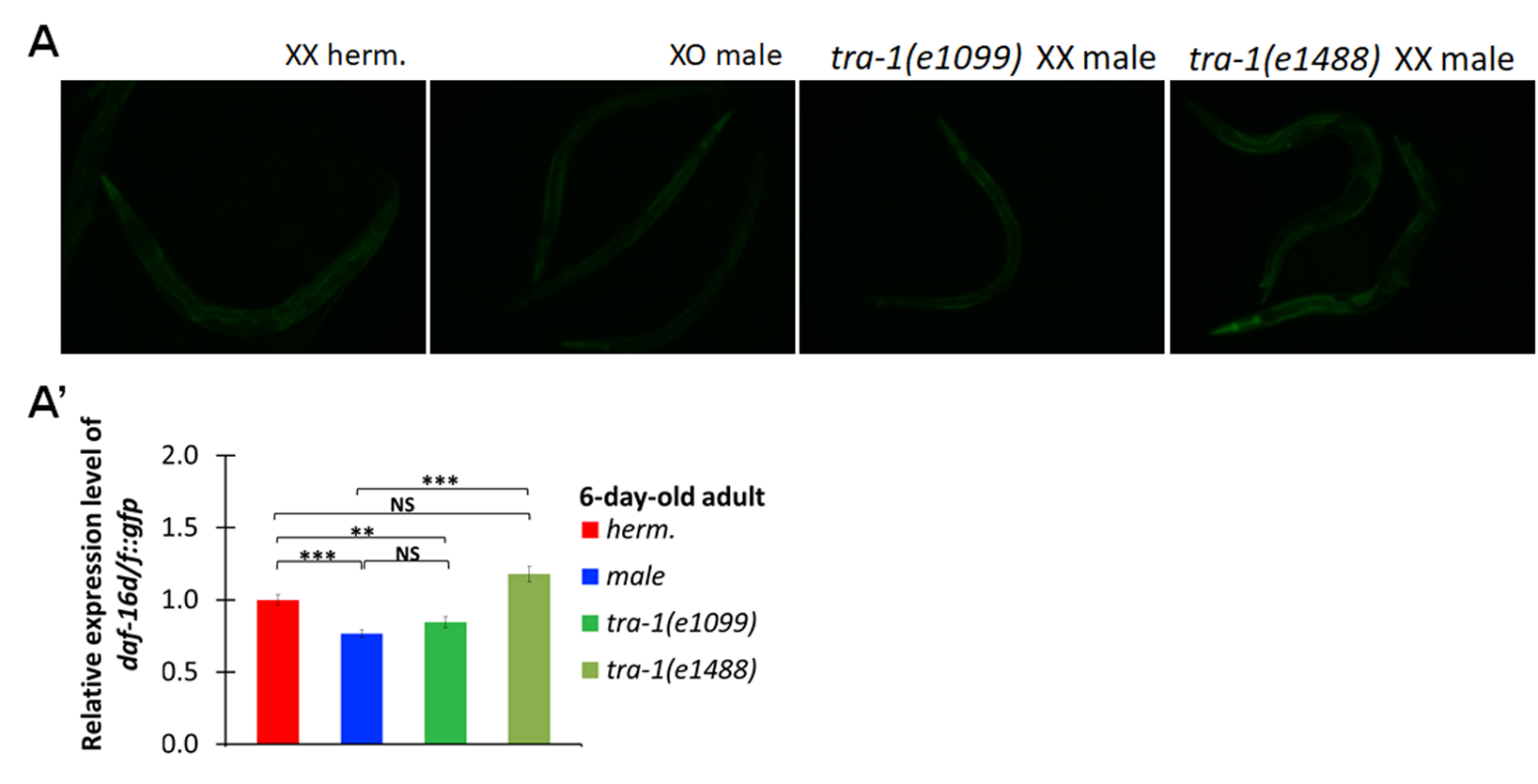
**

**Figure S5. Sex differences in *daf-16d/f* expression are also obvious in aged adults.** (**A**) Fluorescent images showing *daf-16d/f::gfp* expression in wild-type hermaphrodites versus males, as well as in *tra-1(-)* mutant XX males at the 6-day-old adult stage. Pictures were taken with the same exposure time. Gentotypes are indicated. (**A’**) Quantification of *daf-16d/f::gfp* expression levels in different genetic backgrounds. Bars represent ±S.E.M., **: P<0.01, ***: P<0.001; NS: not significant; Mann-Whitney-test (with Bonferroni correction). For statistics and data, see **Table S7**.

**Figure S6.**

**
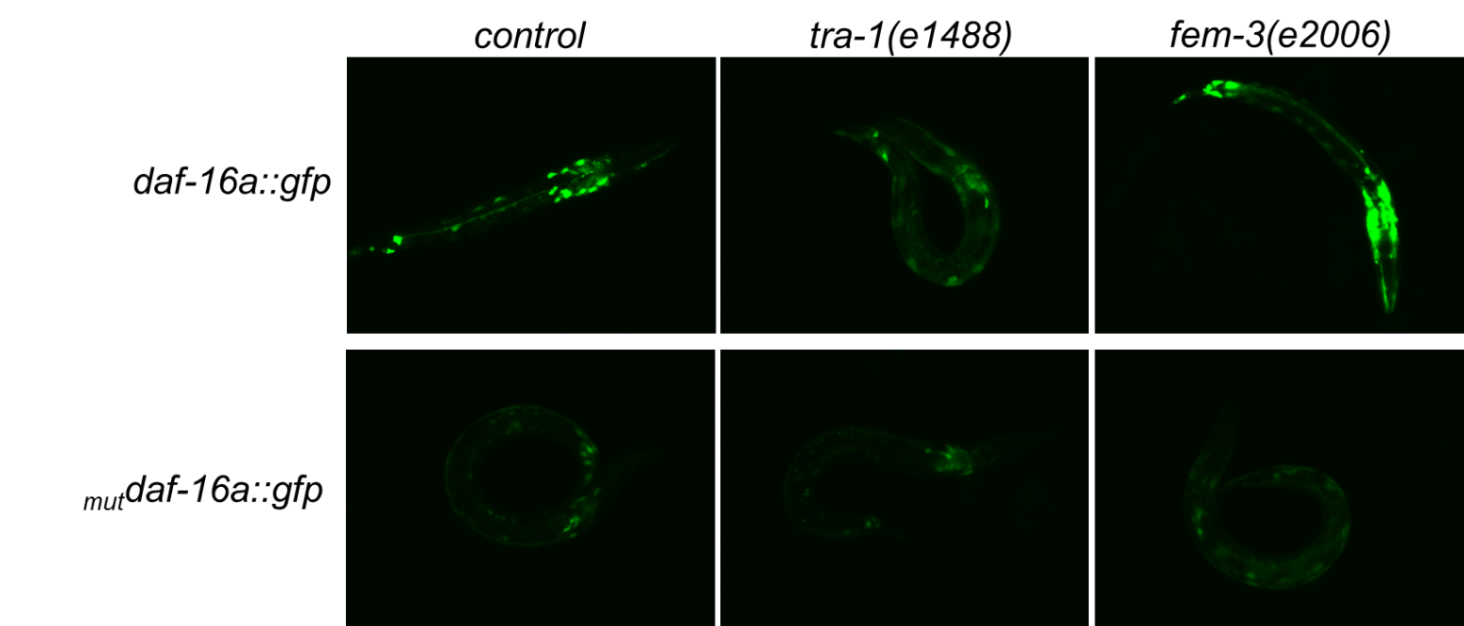
**

**Figure S6. At the L1/2 larval stage, expression of *daf-16a::gfp* is decreased in *tra-1(-)* and increased in *fem-3(-)* mutant backgrounds.** Pictures were taken with the same exposure time (500 ms) and magnification. The activity of *tra-1* and *fem-3* does not influence *_mut_daf-16a::gfp* expression. For statistics, see **Table S2.**

**Figure S7.**


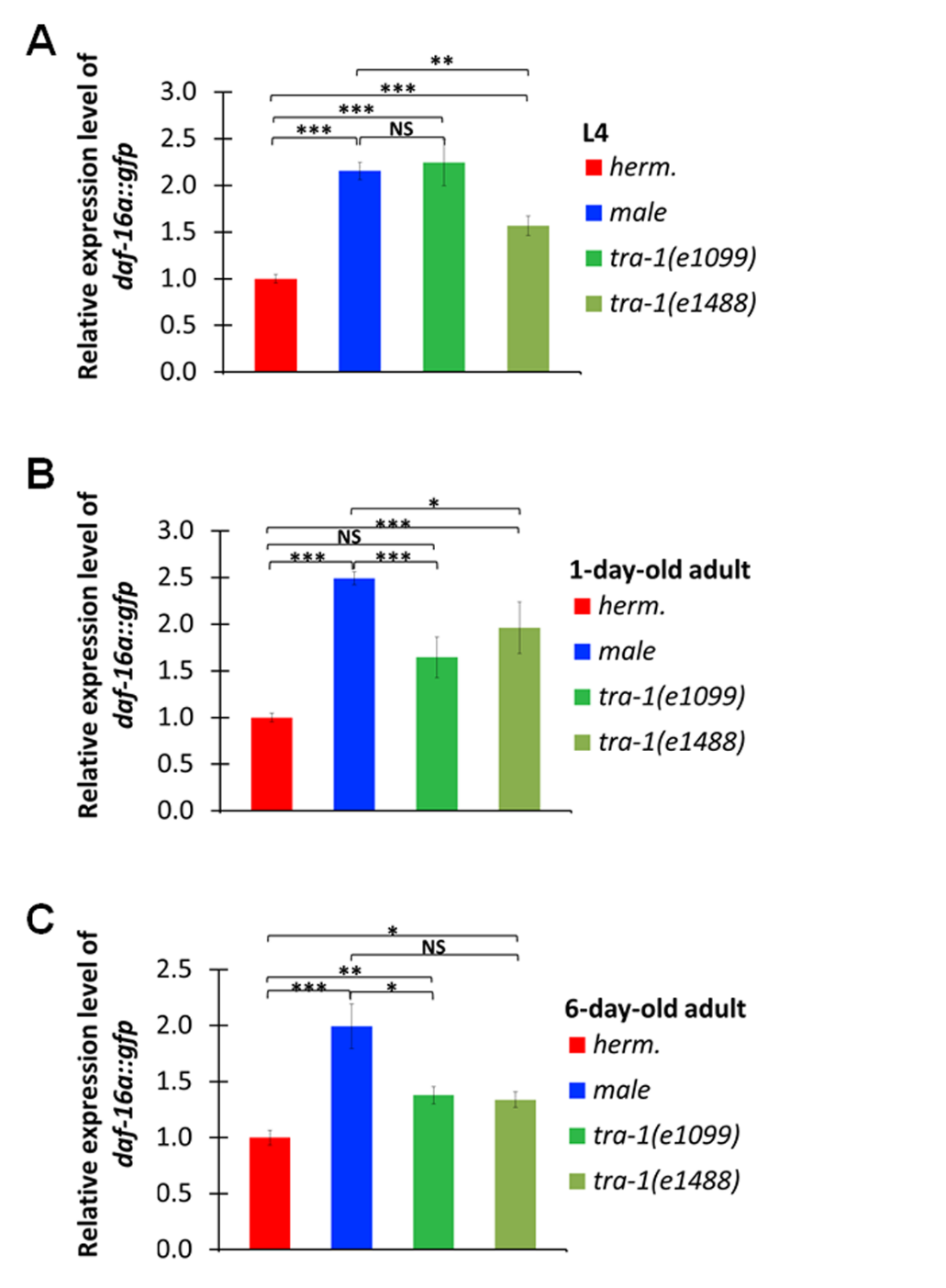


**Figure S7. At late developmental stages and during adulthood, *daf-16a::gfp* expression does not activated by TRA-1.** (**A**-**C**) Relative *daf-16a::gfp* expression levels at the L4 larval, young (1-day-old) adult and aged (6-day-old) adult stages, respectively. Bars represent ±S.E.M., *: P<0.05; **: P<0.01, ***: P<0.001; NS: not significant; Mann-Whitney-test (with Bonferroni correction). For statistics and data, see **Table S8**.

**Figure S8.**

**
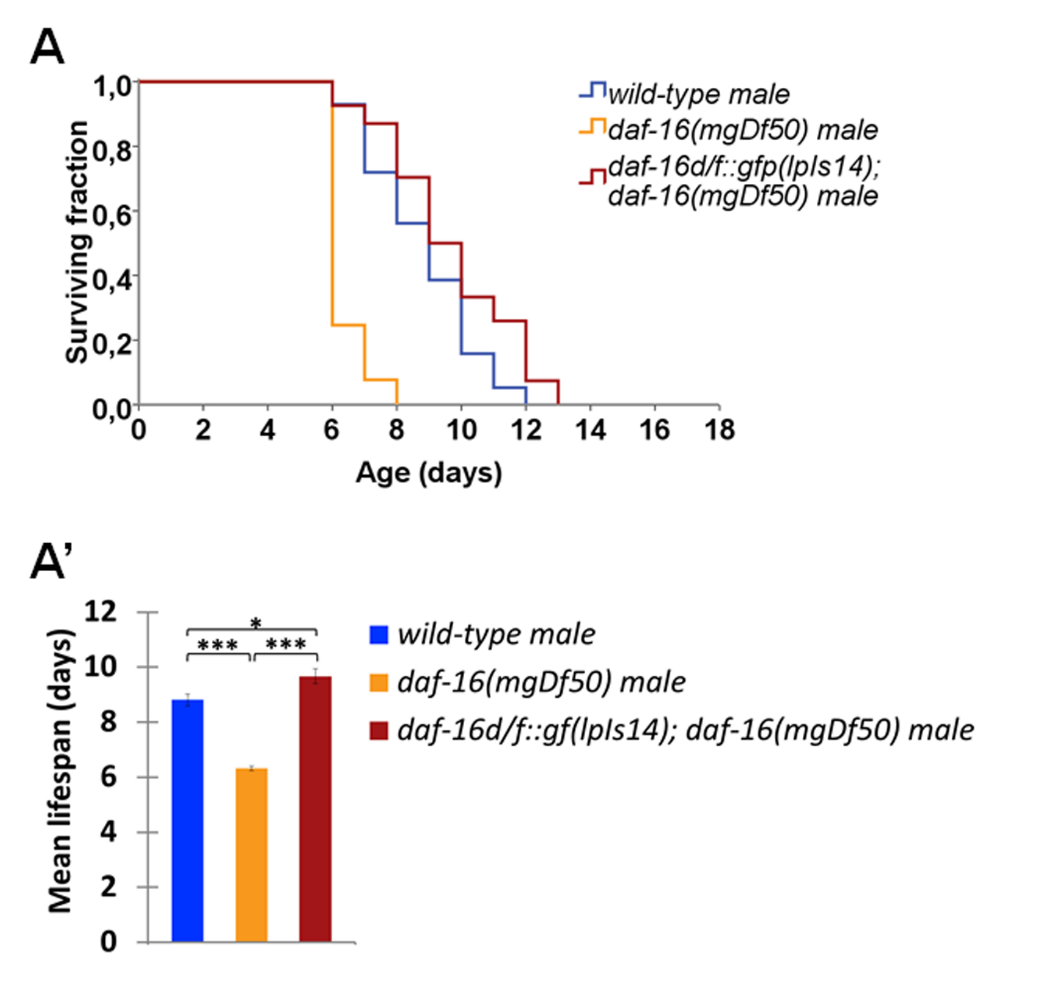
**

**Figure S8.** *lpIs14* **(*daf-16d/f::gfp*) transgene rescues normal life span in *daf-16(-)* mutant males maintained in groups.** (**A**) Life span curve of wild-type males, *daf-16(-)* mutant males and *daf-16(-)* mutant males transgenic for *lpIs14*. *lpIs14* denotes an integrated, full-length *daf-16d/f::gfp* transgene (Kwon *et al.,* 2010). Males were maintained in groups (each test plate contained 30 males but no hermaphrodite). (**A’**) The corresponding mean life span data. The mean life span of transgenic *daf-16(-)* mutant males exceeds that of wild-type males. * indicates P<0.01, *** indicates P<0.001, Log Rank and Independent samples *t*-test with Bonferroni correction. For statistics and data, see also **Table S1**.

**Figure S9.**

**
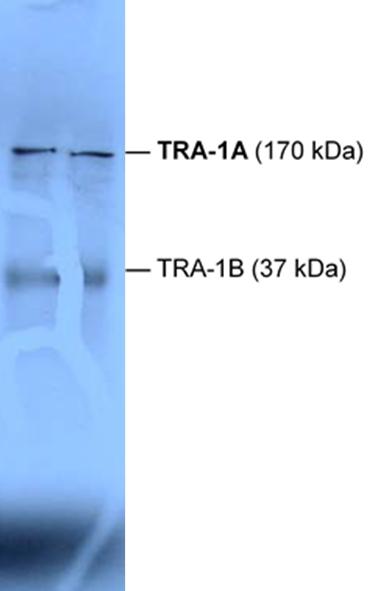
**

**Figure S9. Electrophoretic analysis of *in vitro* synthetized and radiolabeled TRA-1 proteins shows two distinct bands, one at 175 kDa (TRA-1A isoform) and another at 37 kDa (TRA-1B isoform).** TRA-1 proteins were generated from *tra-1* cDNA (kindly provided by David Zarkower, University of Minnesota, US). The larger isoform was also identified at this mass by Segal *et al.* (2001).

**Figure S10.**


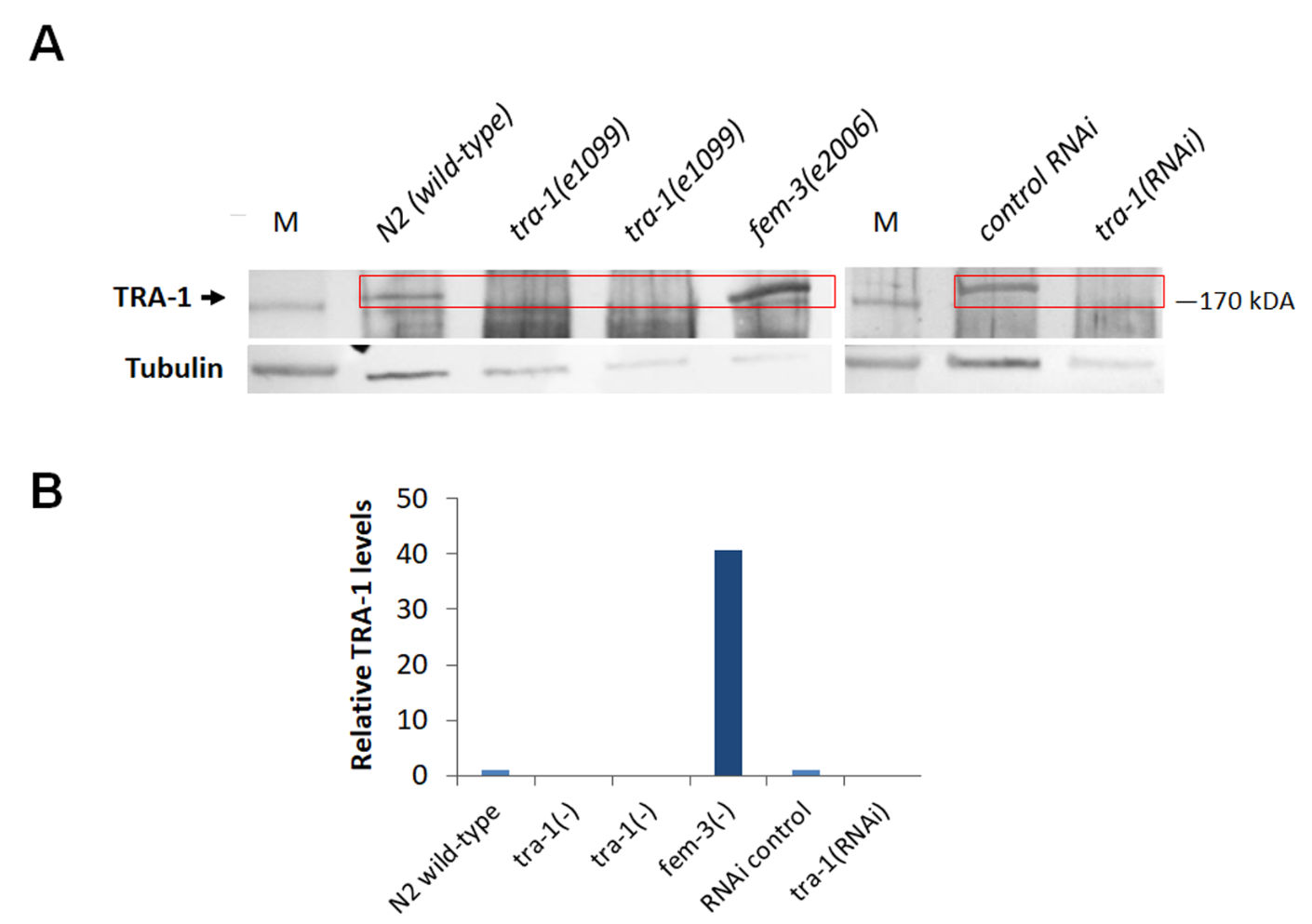


**Figure S10. The specificity of TRA-1 antibody generated in this study (referred as to “Vellai lab” in the text).** (**A**) Western blot analysis showing relative accumulation levels of TRA-1 protein (at 175 kDa; see also Segal *et al.,* 2001) in wild-type (N2), *tra-1* defective and *tra-1* hyperactive genetic backgrounds. TRA-1 disappears in *tra-1(-)* mutant and *tra-1(RNAi)* samples, and is significantly increased in *fem-3(-)* mutant animals, as compared with the corresponding control. α-Tubulin was used as an internal control. M: molecular mass marker. TRA-1-specific bands are highlighted by a red frame. (**B**) Quantification of relative TRA-1 band intensities seen in panel (**A**).

**Figure S11.**


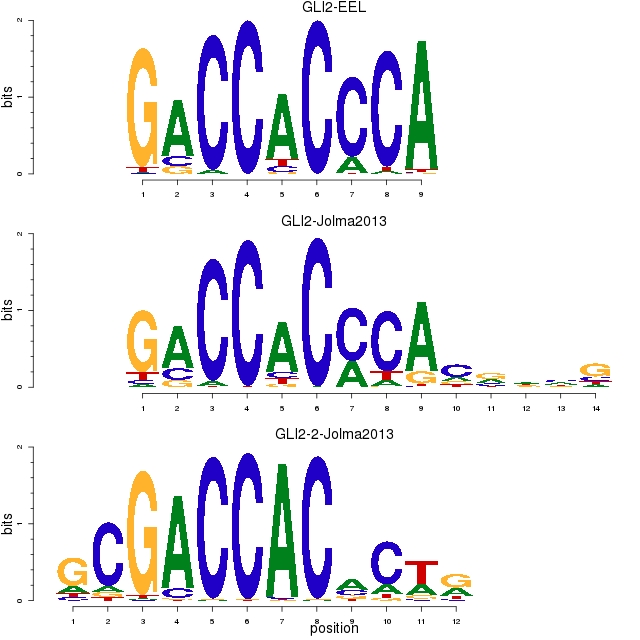


**Figure S11. Sequence logo of the three GLI specific PWMs.** Logos were generated using the seqLogo package of Bioconductor.

**Figure S12.**


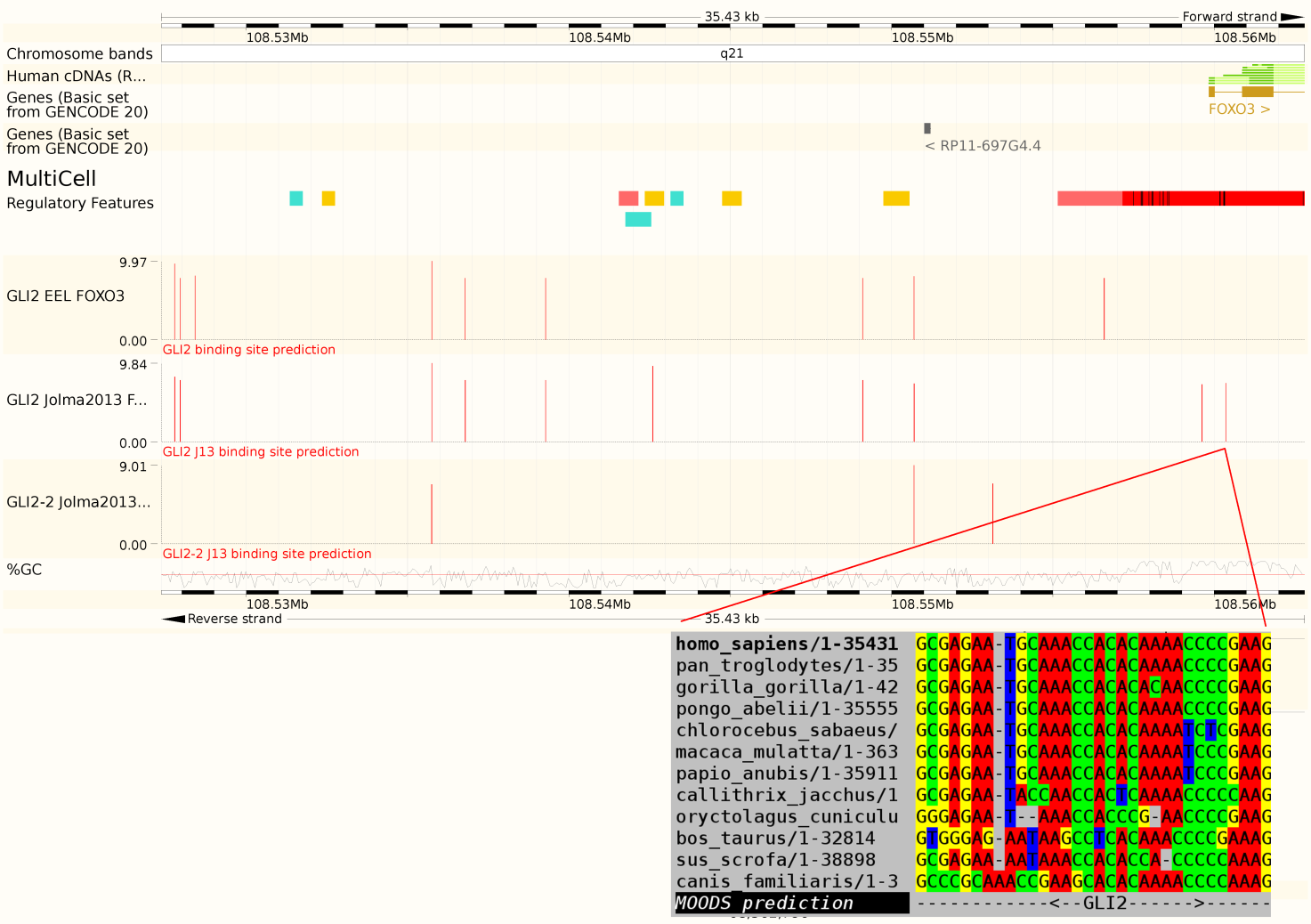


**Figure S12. The genomic region of human FOXO3 with predicted GLI binding sites.** Red lines indicate the position of conserved GLI binding sites identified in the FOXO3 regulatory region. A consensus and highly conserved site is enlarged below, and nucleotides are indicated by different coloring.
